# Exploring neural manifolds across a wide range of intrinsic dimensions

**DOI:** 10.1371/journal.pcbi.1014162

**Published:** 2026-04-03

**Authors:** Jacopo Fadanni, Rosalba Pacelli, Alberto Zucchetta, Pietro Rotondo, Michele Allegra

**Affiliations:** 1 Physics and Astronomy Department, University of Padova, Padova, Italy; 2 Istituto Nazionale di Fisica Nucleare, Sezione di Padova, Padova, Italy; 3 Department of Mathematical, Physical and Computer Sciences, University of Parma, Parma, Italy; 4 Padova Neuroscience Center, University of Padova, Padova, Italy; Salk Institute, UNITED STATES OF AMERICA

## Abstract

The rapid surge in the number of simultaneously recorded neurons demands reliable tools to explore the latent geometry of high-dimensional neural spaces. Within such spaces, neuronal activity typically lies on a subspace or manifold characterized by an intrinsic dimension (ID) that is much lower than the total number of recorded units. The ID can provide immediate information about the neural code, such as the minimum number of encoded variables and the relation between collective and individual neural activity. Existing studies rely on disparate and potentially unreliable ID estimators, which can contribute to conflicting reports of high-dimensional vs. low-dimensional manifolds. Here, we propose a robust and versatile pipeline for ID estimation, exploiting a local version of the full correlation integral estimator (lFCI). Being able to simultaneously cope with high dimensionality and non-linearity, lFCI overcomes some major limitations of common ID estimation methods. We prove the strength and accuracy of lFCI by applying it to synthetic benchmark data by Altan et al., 2019, where other methods typically underestimate the ID. We apply lFCI to study neural manifolds arising in recurrent neural networks trained on the 20 tasks of the well-known ‘cog-Task’ battery. Across tasks and training repetitions, lFCI uncovers a consistently low ID, which we show to be fundamentally related to the task structure. Finally, we apply lFCI to a reference experimental dataset by Stringer et al., 2019, comprising visual responses to a large set of natural images, strongly supporting previous reports that responses are organized in a high-dimensional manifold. lFCI has the potential to shed light on the current debate about the geometry of neural codes, and its dependence on structural constraints and computational goals in biological and artificial neural networks.

## Author summary

The dimensionality, or intrinsic dimension (ID), of neural manifolds is one of the key properties characterizing the collective organization of neural activity at a population level. However, rigorously estimating the ID is difficult: common methods tend to overestimate the ID in the presence of curvature, and underestimate it when large. Exploiting FCI, an ID estimator that is particularly effective in high-dimensional settings, we developed local FCI (lFCI), a pipeline for ID estimation that addresses these limitations. lFCI proves robust when tested against challenging benchmark data. We applied lFCI in two paradigmatic cases: data from artificial recurrent neural networks trained on simple tasks, and experimental data of neural responses to visual stimuli. In both cases, lFCI provides substantial support for widely debated hypotheses - low ID in the case of simple tasks and large ID for visual responses. lFCI could be used to characterize neural manifolds across a wide range of dimensions, offering a useful tool in the current debate about the dimensionality of neural activity.

## Introduction

Traditionally, brain circuits were analyzed by characterizing the functional role of individual units. The advent of large-scale neuronal recordings [1] opened the door to a different approach, focused on understanding the collective activity of all units in terms of representations and computation within abstract spaces [2,3]. Central to this perspective is the concept of *neural manifold* [4,5], the set of configurations visited by the system within the space defined by the activity of all units [6–10]. Visualizing, analyzing, and modeling neural manifolds requires identifying a minimum set of coordinates describing the manifold. The minimum number of coordinates is called the manifold’s *intrinsic dimension (ID)*. The ID encapsulates the complexity of neuronal dynamics, in the classical sense of the relation between the individual units and the whole system [11]. The ID also bounds the possible number of cognitively and behaviorally relevant variables directly encoded by neurons at a population level.

Characterizing the ID of neural manifolds is essential to understand how collective neuronal activity supports the execution of cognitive tasks. Several studies investigating neural manifolds during simple instructed tasks reported a low (≲10) ID [12–17], inspiring a large body of theoretical work aiming to explain how low-dimensional activity can emerge from structural constraints [18–21]. However, many other studies reported a high ID, typically in presence of complex stimulus spaces or loose task structure [22,23]. A thorough comprehension of what determines the geometry of neural manifolds, of which the ID is a key characteristic, is still missing. The specific type of computation or ‘task’ being performed is certainly a crucial factor, but not the only one: for any given task, neural networks may also need to strike an optimal trade-off between conflicting demands such as robustness (which favors low dimensional codes [24]) and ease of readout (which favors high-dimensional ones [25–30]). A clearer understanding demands progress not only in neuronal recording techniques, but also in analysis methodology. In this context, a key gap is the lack of a fully reliable and shared methodology to measure the ID of neural manifolds [31].

Research on neural manifolds often employs linear ID estimation methods [14,15,20,22,23,26,27,32–40], which are based on the spectrum of the covariance matrix and use several criteria to select the number of ‘relevant’ or ‘significant’ dimensions. ID estimates by linear methods generally coincide with the dimension of the (minimal) linear subspace encompassing the manifold [41], rather than the manifold’s actual ID. As a result, they can significantly overestimate the ID in the case of non-linear, curved manifolds, despite the fact that the latter are quite common [42–44]. An alternative to linear methods is provided by geometric ID estimators [45–47], which are ultimately based on the principle that distances in the data follow scaling laws depending parametrically on the ID. The major shortcoming of these methods is that they are not robust to undersampling, requiring a sample size growing exponentially with the ID. As a consequence, they are generally unable to yield proper ID estimates when the ID is large (>rsim10). These shortcomings were highlighted in Ref  [31], where simulated data were used to show that no common method can correctly identify the ID of non-linear, high-dimensional neural manifolds.

Here, we propose a pipeline to evaluate the ID of neural manifolds by leveraging the Full Correlation Integral (FCI) estimator [48]. The major advantage of FCI is its remarkable robustness with respect to undersampling, as it can estimate high IDs from samples whose size scales only linearly with the ID. On the other hand, FCI typically overestimates the ID in the presence of curvature, similarly to linear methods. To overcome this drawback, our pipeline employs a local version of FCI, called *local FCI (lFCI)*. We explore lFCI’s potential by applying it to artificial and real data sets: (i) neural manifolds generated by recurrent neural networks (RNN) trained on the ‘cog-task’ battery [49]. This task set, inspired by real tasks used in neurophysiological studies on non-human animals, involves basic cognitive processes such as working memory, inhibition, and context-dependent integration and it has become a sort of standard [50–54]. The simplicity of the stimulus-response patterns in RNNs is reflected in a strong and consistent expectation about the low dimensionality of the corresponding neural manifolds. (ii) a benchmark dataset of synthetic neural trajectories, embedded in high-dimensional space both linearly and non-linearly [31]. Trajectories are generated from an empirical distribution of firing rates, obtained from multi-electrode array recordings in the macaque primary motor cortex. As shown by Altan et al. [31], common linear and nonlinear estimators fail in the high-dimensional case, underestimating the dimension for ID>rsim10. (iii) real data from calcium imaging of the primary visual cortex of mice [22]. This data set contains neural responses to both large (2,800 images) and small (32 images) stimulus spaces. The authors argued that responses are organized in a very high-dimensional manifold (a widely referenced result). Their conclusions, however, are entirely based on linear dimension estimators. For the cog-task battery, we find that our estimator works better than linear estimators and several geometric estimators, providing consistent estimates for different trainings of the networks. In the benchmark dataset, our estimator performs comparably with the best linear estimator (in the linear embedding case) and performs better than other methods (in the non-linear case). For the real dataset, our conclusions align with those of Stringer et al., supporting the existence of a high-dimensional manifold of visual responses.

This manuscript is organized as follows: in Methods, we provide a review of state-of-the-art ID estimators, including FCI, and illustrate the process to generate the synthetic datasets investigated. In the Results section, we present our pipeline (lFCI) and the results of its application to synthetic and real neural manifolds.

## Materials and methods

### Intrinsic dimension estimation

Consider a set $X=\{{\text{x}}_{1},\ldots ,{\text{x}}_{P}\}$ of *P* points embedded in an ambient space of dimension *N*. Assuming that the points are contained, exactly or approximately, within a manifold or hypersurface of *intrinsic dimension D* < *N*, one can try to estimate *D*. There exists a wide array of ID estimation methods (for a classic review, see [55]). To be suitable for characterizing neural activity manifolds, an estimator should ideally work well for non-linear, curved manifolds and a large range of dimensions, including high dimensions where the manifold is undersampled.

Linear ID estimation methods are based on the spectrum of the covariance matrix of the points. Let $\lambda_{k},\;k=1,\ldots ,N$ be the eigenvalues of the covariance matrix in decreasing order ($\lambda_{1}\geq \lambda_{2}\geq \ldots \geq \lambda_{N}$). The corresponding eigenvectors are commonly termed *principal components (PCs)* [56]. The ID can be determined by assessing the ‘relevant number’ of PCs. The most common method consists in searching for a linear subspace accounting for a large fraction of the total variance $𝒱=\sum_{k=1}^{N}\lambda_{k}$, i.e., identifying the minimum *K* such that


$$ID_{PCA,\alpha }=\min K:\frac{\sum_{k=1}^{K}\lambda_{k}}{𝒱}\geq \alpha$$ (1)

(typical choices are α=0.8,0.9,0.95,0.99). Another method computes the *participation ratio* [57]


$$ID_{PR}=\frac{1}{\sum_{k=1}^{N}(\lambda_{k}/𝒱{)}^{2}}$$ (2)

*ID*_*PR*_ corresponds to an ‘effective number’ of relevant dimensions, as typically $ID_{PR}≃e^{H(\lambda )}$ with $H(\lambda )=\sum_{k=1}^{N}(\lambda_{k}/𝒱)\log(\lambda_{k}/𝒱)$ the entropy of the covariance matrix spectrum. Finally, *parallel analysis (PA)* [58] determines the number of significant PCs through a null model. Surrogate data are constructed by resampling the original data by shuffling each data coordinate independently. For each surrogate dataset, the eigenvalues of the covariance matrix are computed. By considering a large number of shuffles, one obtains a null distribution of eigenvalues. Let $\nu_{\alpha }$ be the (1−α)·100-th percentile of the null distribution (typically, α=0.05). One finally considers as significant all original eigenvalues exceeding this critical value,


$$ID_{PA}=\max K:\lambda_{K}\geq \nu_{\alpha }$$ (3)

Linear methods are appropriate only when the manifold is flat, i.e., it lies on a hyperplane. In the presence of curvature, they can largely overestimate the ID.

Among non-linear methods, the most commonly used are *fractal* [59,60] and *nearest-neighbor* methods [45–47]. Fractal methods are based on the fact that the number of points found within a spherical volume of radius *r* follows an ID-dependent scaling law. The most common example is the Correlation Dimension (CorrDim) method first proposed in the classic paper by Grassberger and Procaccia [59]. CorrDim focuses on the *correlation integral* (the density of points within a given cutoff distance *r*):


$$\rho_{X}(r)=\frac{2}{P(P-1)}\sum_{1\leq i\leq j\leq P}\Theta (r-\|{𝐱}_{i}-{𝐱}_{j}\|)$$ (4)

If the points are sampled from a distribution on a *D*-dimensional manifold, then $\rho_{X}(r)\sim r^{D}$ for r→0. Thus, one can extract the ID by measuring the slope of the linear fit of ρ as a function of *r* in a log-log scale ($D=\lim_{r\to 0}\log\rho_{X}(r)/\log r$). This method can work reasonably well for non-linear manifolds, but is fragile with respect to undersampling, as it requires a number of points scaling exponentially with the ID (“curse of dimensionality”), and thus underestimates high IDs. Nearest-neighbor methods are based on the fact that distances between nearest neighbors obey statistical relations that depend parametrically on the ID. The max-likelihood estimator [45] assumes that the data are distributed on a *D*-dimensional manifold and that the density is uniform in a neighborhood containing the first *K* neighbors of each point. Under this assumption, consider $\xi_{i}=\frac{1}{D}\sum_{k=1}^{K-1}\log(\frac{r_{i,K}}{r_{i,k}})$ where *r*_*i*,*k*_ are the distances of the *k*-th neighbors of point **x**_*i*_ in the data. One can show that


$$\xi_{i}\sim Gamma(k-1,1)$$ (5)

from which one can estimate *D* with a maximum likelihood approach on the empirical $\xi_{i}$. The two-nearest-neighbor (Two-NN) estimator [47] restricts the uniform density assumption to the neighborhood containing only the first 2 neighbors of each point. Under this assumption, one can show that


$$\mu_{i}=\frac{r_{i,2}}{r_{i,1}}\sim Pareto(D)$$ (6)

and one can infer *D* through a non-linear fit of the empirical distribution of $\mu_{i}$. Also nearest neighbor methods incur the curse of dimensionality.

### The FCI estimator

**Fig 1:**
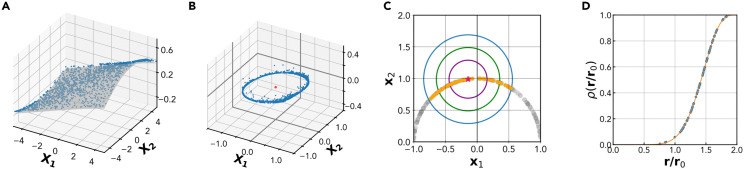
The Full Correlation Integral (FCI) method. Panels A-D illustrate the basic steps of the FCI method for ID estimation. **A** The original *D*-dimensional dataset **B** Centering and normalization of the dataset: the data points are projected on an unitary sphere in *D* – 1 dimensions. **C** The empirical correlation integral of the dataset, $\rho (r/r_{0})$, is measured as a function of the normalized radius *r*/*r*_0_. This amounts to computing how many pairs of points are within a distance *r*. **D** ID estimation: ρ(r) is fitted with its theoretical model given by Eq (7), which depends parametrically on the ID. To obtain the true ID, the estimated ID is increased by one, compensating the removal of one degree of freedom during the normalization step.

The full correlation integral (FCI) estimator proposed by Erba et al. [48] was shown to be remarkably robust to undersampling, being able to correctly estimate large IDs even in the extreme case *P* < *D*. The starting point of the method is provided by CorrDim. To address the curse of dimensionality, Erba et al. made the more restrictive assumption that points are sampled from a rotationally-invariant probability distribution on a *D*-dimensional hyperplane. In this case, after normalization of each point to unit norm, the data must lie on a *D* – 1-dimensional hypersphere (e.g., a circle for *D* = 2, a spherical surface for *D* = 3), and one can exactly calculate the average correlation integral as a function of *r*:


$${𝔼}_{X}[\rho_{X}(r)]=\frac{1}{2}+\frac{\Omega_{D-1}}{2\Omega_{D}}(r^{2}-2{)}_{2}F_{1}\left(\begin{array}{c} \frac{1}{2},\;1-\frac{D}{2}\\ \frac{3}{2} \end{array}\|\;{\left(r^{2}-2\right)}^{2}\right)$$ (7)

where _2_*F*_1_ is the (2,1)-hypergeometric function and $\Omega_{D}$ is the solid angle in dimension *D*. To stress that this analytic formula holds away from the small radius limit of CorrDim, the average correlation integral is named *Full Correlation Integral (FCI)*. With this definition, the FCI, as a function of *r*, has a sigmoidal shape with a slope that depends on *D*. Performing a non-linear regression on the empirical density of neighbors Eq (4) using the FCI Eq (7) results in an algorithm to determine the ID, shown in Fig 1. In detail, the steps are:

1. *Center and normalize the data* (Fig 1A,1B). Calculate the center of mass **b** of the empirical data as $𝐛=1/P\sum_{i=1}^{P}{𝐱}_{i}$ and subtract this quantity from each data point, obtaining a data set centered at the origin. Normalize each sample as ${𝐱}_{i}={𝐱}_{i}/||{𝐱}_{i}||$.
2. *Fit the FCI*. Measure the empirical correlation integral of the dataset at different radii *r* (Fig 1C) and perform a non-linear regression of this empirical neighbors density using the average FCI in Eq (7) as the nonlinear model, and *D* as the free parameter (Fig 1D); increase the estimate by one, D→D+1 to reinstate the degree of freedom removed during the normalization step.

Strong deviations from the predicted functional form of the FCI can be detected by the goodness of fit (GoF), measured as the root-mean-squared deviation of the empirical FCI from its best fit using Eq (7) (in S2 Fig, we report examples of fits with different values of GoF).

By construction, the FCI method is exact for linearly embedded Euclidean spaces sampled with a rotationally-invariant probability distribution. Erba et al. showed that the method is remarkably robust with respect to violations of the rotational invariance assumption. In particular, they showed that FCI estimates work very well even in some cases of strongly non-isotropic distributions (e.g., points sampled from corners of *D*-dimensional hypercubes). In addition, estimates are very robust with respect to undersampling, as correct estimates of D~200 can be obtained from as little as P~20 points.

### Recurrent neural networks performing cog-tasks

RNNs have become a customary tool of computational neuroscience research [61–64]. We trained RNNs to solve the ‘cog-task’ battery first introduced by Yang et al. [49]. For simplicity, we maintained the same RNN architecture and dynamics as originally proposed.

The network has *N*_*rec*_ = 256 recurrent units in the hidden layer coupled with *N*_*in*_=65 input units and *N*_*out*_=33 output units. The input layer is composed of one fixation unit and two rings of 32 units representing two stimuli. The output layer has one fixation unit and a ring of 32 units representing motor output.

In the rate model of network activity, each neuron is represented by a continuous variable $𝐫(t)\in {\mathbb{R}}^{N_{rec}}$ representing the neuron’s membrane potential. The dynamics are described by the equation:


$$\tau \frac{d𝐫}{dt}=-𝐫+f\left(W^{rec}𝐫+W^{in}𝐮+𝐛+\sqrt{2\tau \sigma_{rec}^{2}}\xi \right)$$ (8)

Here, τ=100 ms represents the neuronal time constant, reflecting the slow synaptic dynamics driven by the NMDA receptors. The variable $𝐮\in {\mathbb{R}}^{N_{in}}$ denotes input to the network, while $𝐛\in {\mathbb{R}}^{N_{rec}}$ is the bias or background input. The function f(·) captures the neuronal nonlinearity, and ξ represents *N*_rec_ independent Gaussian white noise processes with zero mean and unit variance, and $\sigma_{rec}=0.05$ quantifies the noise intensity. A typical Softplus function is used, f(x)=log(1+exp(x)), which after re-parametrization is very similar to the neuronal nonlinearity (frequency-current curve) commonly used in neural circuit models. A set of output units **z** read out nonlinearly from the network,


$$𝐳=g\left(W^{out}𝐫\right)$$ (9)

where g(x)=1/(1+exp(−x)) is the logistic function that limits output activities between 0 and 1. *W*^in^, *W*^rec^, *W*^out^ are the input, recurrent, and output connection matrices, respectively. After using the first-order Euler approximation with a time-discretization step Δt, we have


$$\begin{array}{c} {𝐫}_{t}=\left(1-\alpha \right){𝐫}_{t-1}+\alpha f\left(W^{rec}{𝐫}_{t-1}+W^{in}{𝐮}_{t}+𝐛+\sqrt{2\alpha^{-1}\sigma_{rec}^{2}}𝐍\left(0,1\right)\right) \end{array}$$ (10)

Here, α≡Δt/τ, and *N*(0,1) represent the standard normal distribution. We use a discretization step Δt=20 ms. We imposed no constraint on the sign or structure of the weight matrices *W*^in^, *W*^rec^, *W*^out^. The network and training were implemented in TensorFlow [65]. The network receives three types of noisy input,


$$\begin{array}{c} u=\left(u_{fix},u_{mod1},u_{mod2}\right)+u_{noise}\\ u_{noise}=\sqrt{2/\alpha }\sigma_{in}𝐍\left(0,1\right) \end{array}$$ (11)

The input noise strength is set to $\sigma_{in}=0.01$. The fixation input *u*_fix_ is usually at the high value of 1 when the network is expected to maintain fixation, and changes to 0 when the network is supposed to react. The stimulus inputs **u**_mod1_ and **u**_mod2_ represent one-dimensional circular variables characterized by the angle around a circle. This mirrors what happens in typical experiments with primates, involving *directional stimuli and responses* (for instance, the stimulus is a cloud of dots moving in a given direction, and the animal is asked to move the arm in the same direction). There are 32 units in each of the corresponding stimulus rings, with their preferred directions evenly distributed from 0 to 2π ($\psi_{i}=2\pi \cdot i/32=\pi /16\cdot i$). For unit *i*, which has a preferred direction $\psi_{i}$, its activity in response to a stimulus at direction ψ is given by


$$u_{i}=\gamma \cdot 0.8\exp\left[-\frac{1}{2}{\left(\frac{8\left|\psi -\psi_{i}\right|}{\pi }\right)}^{2}\right],$$ (12)

where γ represents the stimulus intensity. For multiple stimuli, input activities are summed.

The tasks used here are the same as those used by Yang et al. [49]. They selected 20 interrelated tasks, representing common tasks used in neurophysiological studies on nonhuman animals, and useful to understanding basic cognitive processes such as memory-guided response, simple perceptual decision making (DM), context-dependent DM, multisensory integration, parametric working memory, inhibitory control, delayed match-to-sample and delayed match-to-category tasks. The tasks can be divided into three families: the Go, Decision Making (DM), and Matching families.

#### Go family.

In this family, a single stimulus is randomly shown either in modality 1 or 2, and the response should be in the same direction of the stimulus. This family includes the forward-Go (Fd-Go), in which the network should respond when the fixation cue goes off; the reaction time Go (RT-Go), in which the network should respond as soon as the stimulus appears; the delay go (Dly-Go), in which the stimulus is turned off before the go and the network has to respond only after the fixation cue goes off. In the ‘anti’ versions of the tasks (Fd-Anti, RT-Anti and Dly-Anti), the network should respond in a direction opposite to that of the stimulus.

#### DM family.

In this family, two stimuli are shown simultaneously and are presented till the end of the task. A stimulus is drawn randomly on the whole circle while the other is drawn uniformly between 90° and 270° away from the other. The network has to respond in the strongest of the two directions. The family includes DM1 and DM2 where the two stimuli are presented only in modality 1 (respectively, 2); Ctx DM1 and Ctx DM2 in which the network has to consider only the two stimuli presented in modality 1(2) ignoring those in the other modality; MultiSensory DM in which the network has to respond in the direction of the stimulus that has a stronger combined strength in modalities 1 and 2. In the delayed versions of the tasks (Dly DM 1, Dly DM 2, Ctx Dly DM 1 and Ctx Dly DM 2), the two stimuli are not contiguous in time. They are both shown briefly and are separated by a delay period. Another short delay period follows the offset of the second stimulus before the response.

#### Matching family.

In this family, two stimuli are presented consecutively in modality 1 or 2 and separated by a delay period. The response depends on whether or not the two stimuli “match”. This family includes the delayed-match-to-sample (DMS), delayed-non-match-to-sample DNMS, delayed-match-to-category (DMC) and delayed-non-match-to-category DNMC tasks. In DMS and DNMS, two stimuli match if they point towards the same direction; in DMC and DNMC, if their direction belongs to the same ‘category’, the first category ranging from 0 to 180°, the other from 180 to 360°. In DMS and DMC the network has to respond towards the second stimulus when there is a match, and fixate otherwise; in DNMC and DNMS, it has to respond towards the second stimulus when the two stimuli are not matched, and fixate otherwise.

### Synthetic neural recordings

Altan et al. [31] created synthetic data to investigate the performance of ID estimators on neural manifolds. In particular, they constructed synthetic data of known dimensionality, mimicking the properties of multi-electrode neural activity recorded in the primary motor cortex of macaques. Data generation starts by extracting d×M samples from an empirical distribution of firing rates obtained from multi-electrode array recordings of the macaque primary motor cortex [66] with a binning of 50 *ms*. Samples are extracted randomly across all recorded neurons and time bins, resulting in temporally uncorrelated samples. The *d*-dimensional data are then temporally smoothed (with a Gaussian kernel of s.d. 50 *ms*) and multiplied by a N×d mixing matrix with entries randomly selected from a Gaussian distribution of zero mean and unit variance. This produces a dataset *X* composed of *M* samples of dimension *N*. This embedding step does not change the intrinsic dimension *d*. The value of *d* ranges from 3 to 40, while *N* = 96 is similar to the number of electrodes in multi-electrode measurements.

In order to produce a dataset with strongly non-linear properties, an exponential nonlinearity is computed on each of the *N* variables in *X*, yielding a data set


$$Y=f(X)=\frac{e^{\alpha X}-1}{e^{\alpha }-1}$$ (13)

where α determines the degree of nonlinearity. Also this non-linear embedding procedure preserves the ID. However, since the transformation is not an isometry (it does not preserve distances), it may significantly distort neighborhood relations (i.e., points that are near before the transformation may be mapped to points that are far apart), and for a finite sampling retrieving the correct ID may be significantly hard.

### Responses to visual stimuli in mouse V1

We reanalyzed the data from [22], which were made available through Figshare [67]. For detailed methods we refer to the original study [22]. Here we provide a brief summary of the salient elements. *Animals.* While the dataset includes data from 8 mice, here we restricted attention to one animal for which responses to several stimulus sets were available. The mouse (‘M170714_MP032’) was bred to express tdTomato in inhibitory neurons, and GCaMP6s was expressed virally, indentifying excitatory neurons by lack of tdTomato expression. *Imaging* In each of 4 separate recordings, neural activity was recorded through a two-photon microscope using multi-plane acquisition with planes 30 to 35μm apart; 10 or 12 planes were acquired sequentially, scanning the entire stack repeatedly at 3 Hz or 2.5 Hz. The mouse was free to run on an air-floating ball; a field of view was selected such that ~10,000 neurons could be observed, with clear calcium transients and a retinotopic location. In total, 10,075±1,453 (mean ± s.d.) neurons were obtained in the recordings. *Stimuli*. We analyzed four separate recordings. In two recordings, stimuli consisted of different normalized images from the ImageNet database [68], with 2,800 different images used in total (the researchers manually selected images that had a mix of low and high spatial frequencies and that did not consist of more than 50% uniform background. All images were uniformly contrast-normalized by subtracting the local mean brightness and dividing by the local mean contrast). The 2,800 natural image stimuli were displayed twice in a recording in two blocks of the same randomized order. All stimuli were presented for 0.5 s with a random interstimulus interval between 0.3 and 1.1 s consisting of a gray-screen. In one recording, a smaller set of 32 images from the same set was presented in a randomized order 114 times, with similar presentation parameters. Finally, in one recording drifting gratings of 32 directions, spaced evenly at 11.25°, were presented 117 times each, lasting 0.5 s each, and with a gray-screen inter-stimulus interval between 0.3 and 1.1 s. The spatial frequency of the stimuli was 0.05 cycles per degree and their temporal frequency was 2 Hz. *Preprocessing.* Calcium movie data were processed using the Suite2p toolbox to estimate spike rates of neurons. Underlying neural activity was estimated using nonnegative spike deconvolution. These deconvolved traces were normalized to the mean and SD of their activity during a 30-min period of gray-screen spontaneous activity. For further detail, please see the original study. Stimulus responses were computed from the first two frames acquired after stimulus onset for each plane. All analyses done in this paper were performed on the preprocessed data available on figshare [67].

### ID and dimensionality reduction

Once an estimate of the ID is achieved, it can be coupled with a dimensionality reduction method to obtain a lower-dimensional representation of the data. For nonlinear manifolds, there are several well-established methods to do so. For instance, Locally Linear Embedding (LLE) [69] assumes the data lie on a smooth manifold of dimension *D* and that each point can be approximated by a linear combination of its nearest neighbors. For each data point **x**_*i*_, LLE computes reconstruction weights *w*_*ij*_ by minimizing


$$\min_{w_{ij}}||{𝐱}_{i}-\sum_{j\in 𝒩(i)}w_{ij}{𝐱}_{j}|{|}^{2}\;\text{subject to}\;\sum_{j}w_{ij}=1$$

These weights capture the local geometry of the data. LLE then finds a *D*-dimensional embedding **y**_*i*_ that preserves these relationships by minimizing


$$\min_{{𝐲}_{i}}||{𝐲}_{i}-\sum_{j}w_{ij}{𝐲}_{j}|{|}^{2}$$

which leads to an eigenvalue problem. The resulting embedding preserves local neighborhood structure. Isomap [70] is another common nonlinear dimensionality reduction technique, similar to LLE in spirit. Isomap first uses a graph representation of the data to approximate geodesic distances on a manifold. It then applies classical multidimensional scaling on the geodesic distances to project the data in *D* dimensions. Recently, an extension of Isomap working for high-dimensional data sets has been proposed [71]. An alternative to classical methods is offered by deep autoencoders [72] - neural networks that learn compact, low-dimensional representations of data by training an encoder–decoder architecture to reconstruct the input. By stacking multiple nonlinear layers, they can capture complex, hierarchical structures that other methods cannot.

## Results

### The local FCI pipeline

We start by presenting our pipeline for intrinsic dimension (ID) estimation. Our method builds on the Full Correlation Integral (FCI) estimator [48] (Methods). FCI assumes an isotropic point distribution on a hyperplane centered on the center-of-mass of the points. While FCI is exceptionally robust in high dimension compared to other methods, it can give suboptimal performance when its assumptions are violated, i.e., in the presence of curvature or violations of the isotropy assumptions. Ref  [48] provided preliminary evidence that both limitations could be addressed by performing ID estimates locally on selected neighborhoods on the manifold. Indeed, i) local neighborhoods approximate the tangent plane to the manifold and are approximately flat and ii) the local distribution of points is typically approximately isotropic, unless a very strong density gradient is present. However, Ref  [48] did not turn this into a systematic procedure for local ID estimation. Here, we leverage such insight to propose a systematic and robust procedure for ID estimation.

**Fig 2:**
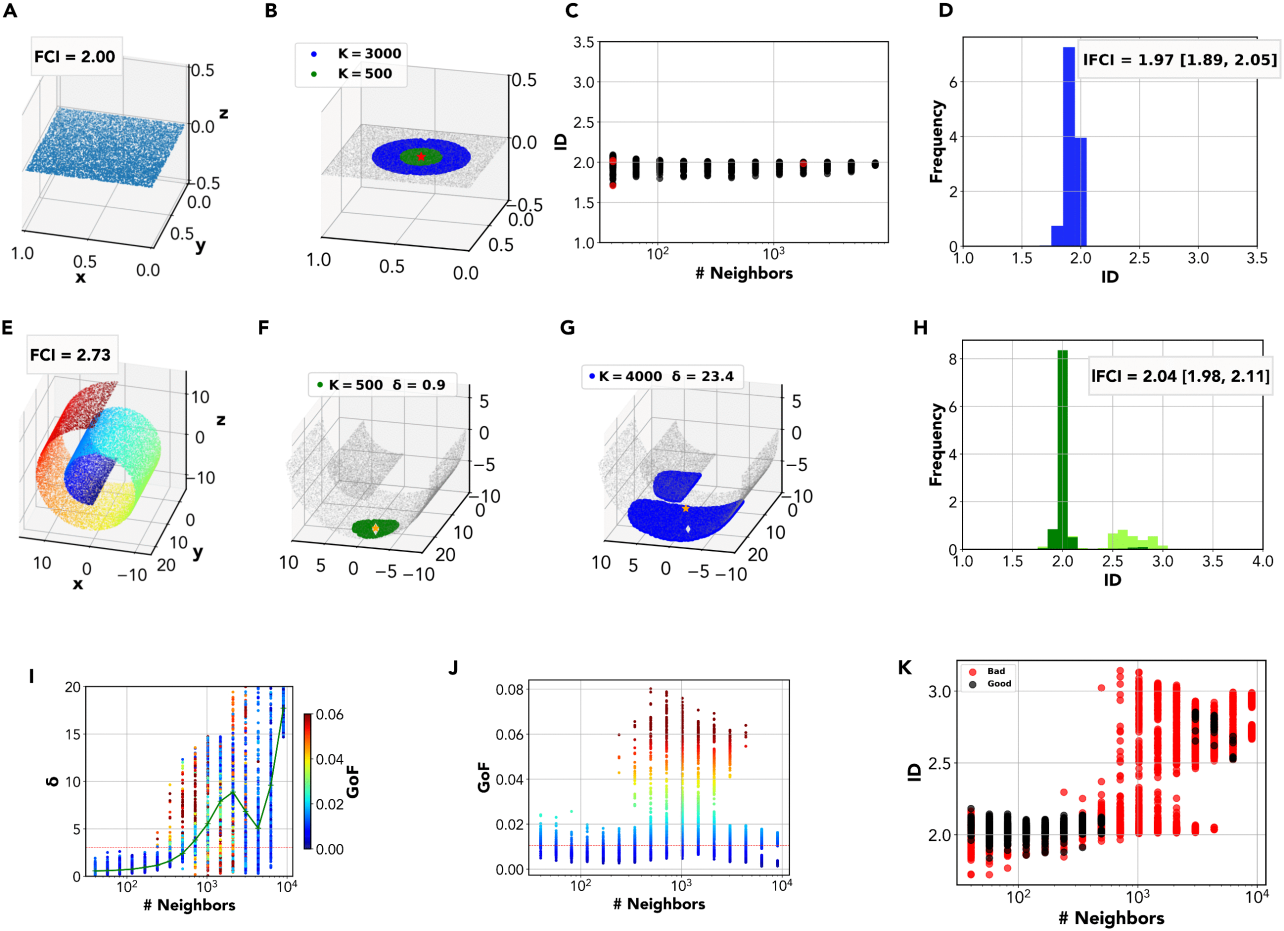
The Local FCI method. **A** A dataset with uniform distribution on a plane, *ID* = 2. FCI correctly identifies the ID of the dataset, yielding *ID*_*FCI*_ = 2.00. **B** Local ID estimates can be computed using FCI on neighborhoods of increasing size *K*. **C** In the *multiscale ID plot*, local ID estimates are shown as function of the neighborhood size ***K*.** Estimates are colored according to their reliability, as assessed by Goodness-of-Fit (GoF). **D** All reliable local ID estimates for different *K* are collected in the *local ID histogram*. The mode of local ID estimates (the peak of the histogram) yields a single overall ID estimate. We call this ID estimation method the *local FCI estimation* (lFCI) method. For the 2-D plane, we obtain *ID*_*lFCI*_ = 1.97 [1.89,2.05]. **E** A dataset with points uniformly distributed on a Swiss Roll. Even though the ID is 2, FCI gives *ID*_*FCI*_ = 2.73. The global FCI overestimates the ID due to the curvature of the manifold. **F** Neighborhoods of small size ($K≲10^{3}$ approximate well the local tangent plane to the manifold. The center-of-mass is located within the neighborhood (low δ). **G** As the neighborhood size increases, neighborhoods depart from the local tangent plane, becoming curved and even possibly disconnected. As a result, the center-of-mass is located outside of the neighborhood (high δ). **H** The local ID histogram for the Swiss Roll shows a prominent peak at 2. We obtain *ID*_*lFCI*_ = 2.04 [1.98, 2.11]. **I** Distribution of the ratio between distance of the center-of-mass and the average distance between points, δ, for the different neighborhoods sizes. The value of δ increases at the increasing of the neighborhood size and, around $K>rsim10^{3}$, most of the neighborhoods become curved with δ>2 (δ=2 is the red line in the plot). In green the median value of δ. **J** Distribution of the *GoF* values for the different neighborhoods sizes. The red line is the threshold chosen as the smallest 99^th^ percentile of the *GoF* distributions for the different neighborhoods sizes. *GoF*_*thr*_ = 0.01. The neighborhoods with size $K\sim 10^{3}$ are the less reliable. **K** In the *multiscale ID plot* for the Swiss Roll, we observe a sharp transition: for $K≲10^{3}$, local ID estimates are close to 2, while for larger *K* they approach 3. However, we can discriminate bona fide estimates (δ<2 and *GoF* < 0.01), shown in black, from unreliable ones (δ>2 or *GoF* > 0.01), shown in red. For $K>rsim10^{3}$, most estimates are unreliable.

The main limitation of FCI can be illustrated with a simple example. When points are sampled from a uniform distribution on a two-dimensional plane, FCI yields a very accurate estimate, *D* = 1.97 (Fig 2A). When points are uniformly sampled on a Swiss roll - a curved two-dimensional surface embedded in a three-dimensional space - FCI overestimates the dimension, yielding *D* = 2.73 (Fig 2E). Let us try to address this limitation by exploiting local ID estimates. For any given point **x**_*i*_ in the dataset, one can consider its first *K* neighbors,


$${ℬ}_{K}({𝐱}_{i})=\{{𝐱}_{j}\text{ such that }||{𝐱}_{j}-{𝐱}_{i}||<r_{i,K}\}$$

where *r*_*i*,*K*_ is the distance of the *K*-th neighbor. Local neighborhoods of different size are shown for the plane in Fig 2B. By restricting the ID estimation to points within the neighborhood ${ℬ}_{K}({𝐱}_{i})$, one obtains a local ID estimate *D*_*i*,*K*_. One can randomly subselect M≫1 points and consider neighborhoods of increasing size *K* centered at each of these points, computing *D*_*i*,*K*_ for each point and neighborhood size. Estimates *D*_*i*,*K*_ as a function of the scale *K* can be collectively organized in a *multiscale ID plot*. This is shown for the 2-D plane in Fig 2C: estimates at all scales give values in the narrow range [1.7,2.2]. By collecting all local estimates for different *K* in a single *local ID histogram*, we obtain a sharp peak around the expected value, 2 (Fig 2D).

When replicating this procedure on the Swiss roll, we observe that estimates *D*_*i*,*K*_ remain around 2 for small neighborhood sizes ($K≲10^{3}$), while they progressively approach the value 3 for larger neighborhoods (Fig 2H). This is rather unsurprising: for sufficiently small *K*, the neighborhoods remain nearly flat, approximating the local tangent plane of the manifold, and local ID estimates correspond to the manifold’s proper ID; for larger *K*, the neighborhoods become curved and the estimates converge to the standard global FCI estimate. This suggests that *a proper ID estimate could be obtained from the collection of local estimates, removing curved neighborhoods from the analysis*.

Two metrics can track the departure of local neighborhoods from the tangent plane. The first is the position of the neighborhood’s *center-of-mass*, ${𝐛}_{i,K}=\frac{1}{K}\sum_{j}{𝐱}_{j},\;{𝐱}_{j}\in {ℬ}_{K}({𝐱}_{i})$. For a flat neighborhood, the center-of-mass **b**_*i*,*K*_ lies among the points of the neighborhood: as a result, the center-of-mass’s distance from the closest point in the neighborhood is of the same order as the typical distance between the nearest neighbors within the neighborhood. For a curved neighborhood, instead, **b**_*i*,*K*_ is displaced from the neighborhood (for an extreme case, think of a spherical surface: its center-of-mass is the sphere’s center, located far off all surface points). This behavior is effectively captured by the metric


$$\delta_{i,K}=\frac{\min_{j}||{𝐛}_{i,K}-{𝐱}_{j}||}{\frac{1}{K}\sum_{j}\min_{k}||{𝐱}_{j}-{𝐱}_{k}||}\;\{{𝐱}_{j},{𝐱}_{k}\in {ℬ}_{K}({𝐱}_{i})\}$$ (14)

which is simply the distance between the center-of-mass and the closest neighborhood point, normalized by the average nearest-neighbor distance in **b**_*i*,*K*_. For flat neighborhoods, we expect δ~1, while curved neighborhoods will give δ>1. The second metric is the FCI Goodness-of-Fit (GoF) (Methods), measured as the average discrepancy between the empirical FCI curve and its fit (i.e., larger values correspond to worse fit quality). Because the center-of-mass is located off the manifold, the points are not distributed isotropically around it, as assumed by the FCI model. Therefore, the fit quality is often poor.

The effectiveness of these metrics can be exemplified by considering two neighborhoods of different sizes on the Swiss Roll. In Fig 2F, we show a neighborhood with *K* = 500. The neighborhood approximates the tangent plane at **x**_*i*_ and it is nearly flat, yielding $\delta_{i,K}=0.9$. Correspondingly, we obtain a good fit (GoF = 0.003) and an accurate ID estimate *D*_*i*,*K*_ = 2.04. In Fig 2G, we show a larger neighborhood with *K* = 4000. The neighborhood strongly deviates from the tangent plane at **x**_*i*_, yielding $\delta_{i,K}\sim 15$. Correspondingly, we obtain a much worse fit (GoF = 0.022) and an excessive ID estimate *D*_*i*,*K*_ = 2.65. The just exemplified behavior is systematic, as shown in Fig 2J. For small neighborhoods (K≲500) we invariably obtain δ~1. For larger ones (K>rsim500), δ progressively grows and the fit quality degrades.

Extensive tests on data sampled on flat manifolds showed that δ remains in the range [0,2] with near certainty (S1 Text and S1 Text and S8 Fig). Therefore, δ>2 is a robust and conservative criterion for curvature detection, and we can consider local ID estimates as unreliable (due to curvature) if δ>2. The range of GoF values, instead, can widely vary within a dataset, even a flat one. Neighborhoods of specific sizes *K* can yield much worse GoF than others not only because they are more curved, as in the case of the Swiss Roll, but also because isotropy violations become more prominent (S1 Text and S9 Fig). When this wide variability occurs, in the sake of accuracy it is reasonable to restrict attention to the best local estimates. To this aim, we may identify a ‘reference’ GoF distribution by considering the size *K* at which the best (lowest) GoF is observed, and consider local estimates as unreliable (due to comparatively bad fit quality) if the corresponding GoF lies out of this distribution. In the multiscale ID plot for the Swiss Roll, (Fig 2K) we display reliable estimates in black and unreliable ones in red. Nearly all reliable ID estimates are around 2; conversely, excessive (~3) estimates are nearly always spotted as unreliable. Congruently, while the local ID histogram is bimodal, with a peak at 2 (determined by small neighborhoods) and a peak at ~3 (determined by large neighborhoods), excluding unreliable estimates suppresses the peak at 3 (Fig 2H).

We thus propose the following *local FCI (lFCI)* pipeline:

1. compute local estimates *D*_*i*,*K*_ on randomly selected neighborhoods with different values of *K*.
2. identify the scale $\bar{K}$ yielding estimates with the lowest GoF, and compute the 99% percentile γ of the GoF distribution for neighborhoods of size *K*.
3. Discard estimates corresponding to sizable curvature ($\delta_{i,K}>2$) or comparatively poor fit quality (GoF >γ)
4. Collect all local ID estimates in a histogram and take its highest peak $D^{*}$ as the best estimate of the overall ID.
5. Specify a plausible range for ID by reporting the 10th and 90th percentile of the local ID estimates.

This pipeline is expected to work well for sufficiently well-sampled Riemannian manifolds, such that local neighborhoods will approximate an isotropic sampling on the manifold’s tangent plane.

The computational complexity of lFCI scales as $𝒪(N^{2}DM)$ (S1 Text). As *N* and *D* are fixed by the data set, the major free parameter entering the computational cost is the number of centers, *M*. For our results, we used *M* = 100 centers, which ensures an approximate covering of the whole dataset even when using small neighborhoods (K~100). Running the code on a Desktop workstation with 8 Intel i7-8550U cores for a dataset of 10000 points took ~5 minutes.

### ID of neural manifolds of recurrent neural networks performing the cog-task battery

**Fig 3:**
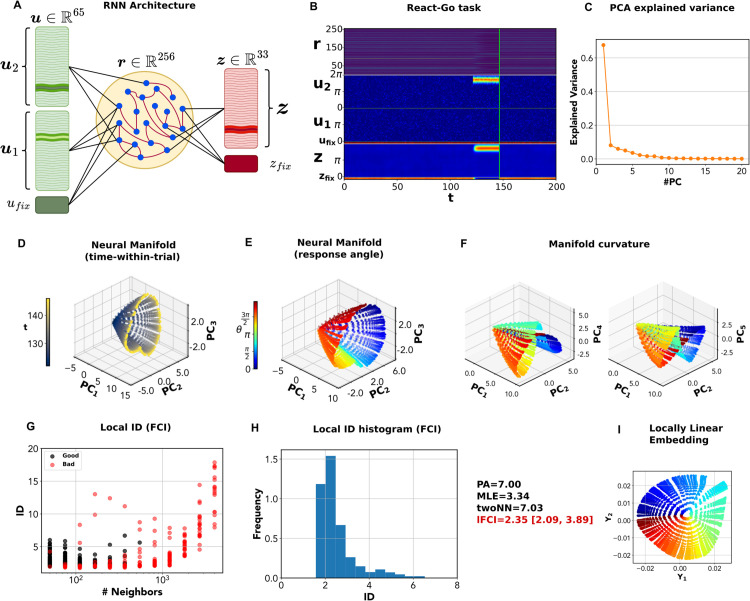
Intrinsic dimension of the neural manifold in the React-Go task. **A** Schematic structure of the RNN used for the Cog-Task battery. The network has 65 input units: two sets of 32 channels each, $u_{1},\;u_{2}$, respectively encode two angular stimuli that jointly determine the network’s response; the fixation input *u*_*fix*_ instructs the network on when to respond. The recurrent layer has 256 units. The output layer has 33 units: 32 units represent an (angular) motor response, *z*, while the last unit represents a ‘fixation’ response *z*_*fix*_. **B** Input, output and network activity for the RT-Go task. In this task, a single angular stimulus is presented, and the network should immediately give a response in the same direction. **C** Variance explained by successive principal components (PCs) of the network activity. **D** Projection of the network activity on the first three principal components. The color code stands for time-within-trial (time from trial onset). *PC*_1_ is nearly aligned with time-within-trial. Starting from the origin (null activity), the trajectories reach a ‘ring’ of attractor points (yellow). **E** Projection of the network activity on the first three principal components. The color code stands for response angle. At any time-within-trial, responses lie on a ‘ring’ encoding the response angle. **F** Projection onto different principal components. The manifold is not entirely contained in the space spanned by the first 3 principal components, but ‘stretched’ along several orthogonal axes. **G** The multiscale ID plot shows ID estimates as a function of *K*, in black (reliable estimates) or red (unreliable ones). At high *K* > 500, all estimates become unreliable. **H** The local ID histogram shows a narrow peak around 2. Estimating the mode of the distribution yields *ID*_*lFCI*_ = 2.35 [2.09, 3.89]. A linear ID estimation method, parallel analysis (PA), finds *ID*_*PA*_ = 7. The classical geometric maximum-likelihood estimator (MLE) yields *ID*_*MLE*_ = 3.34, while Two-NN yields *ID*_*TwoNN*_ = 7.03, a severe overestimation due to its high sensitivity to local noise. **I** We used locally linear embedding (LLE) to find a global low-dimensional representation of the neural manifold for RT-Go. LLE flattens the manifold onto a circle spanned by a radial coordinate, representing time-within-trial, and an angular coordinate, representing response angle.

We trained recurrent neural networks (RNNs) to perform the ‘cog-tasks’ battery [49] (Fig 3A). For each task, we trained *M* = 10 networks. After training, each RNN performed *N* = 200 trials of the task. For each network, we defined the neural manifold as the set of configurations (in the space of activities of the recurrent layer neurons) visited by the network across the *N* trials. We characterized each manifold with common ID estimators.

Before analyzing how the manifold’s ID varies across tasks and trainings, we provide a detailed analysis of an exemplary case: the neural manifold of a RNN performing the React-Go task, where the network must respond with an output in the same direction of the input stimulus (Fig 3B). We first performed principal component analysis. The first principal component explains 64% of the variance, while four subsequent ones explain 5–10% (Fig 3C). As a result, parallel analysis finds *ID*_*PA*_ = 7. In the space defined by the first 3 principal components, the manifold appears to be topologically equivalent to a cone (Fig 3D and 3E). The cone axis is roughly aligned to the first principal component, while for fixed values of the first principal component, the points describe a (deformed) circle in the space spanned by the second and third principal component. In Fig 3D, points are colored according to time-within-trial (*t* = 0 start of trial; *t* = 152 end of trial); in Fig 3E, according to response angle. These representations allow a ready explanation for the manifold’s shape. Before the stimulus presentation, the network is silent (**r** = 0). This configuration corresponds to the vertex of the cone. As the stimulus is presented, the network reacts by reaching a set of attractor states that depend on the response angle and approximately describe a circle. These attractor points are not reached immediately, hence trajectories span a ‘conical’ surface. Importantly, the surface does not lie exactly in the linear space spanned by the first three principal components. In fact, the cone is ‘tilted’ in several dimensions. This is shown in Fig 3F, where we replace the third principal component with successive principal components. As a result, linear estimates of the ID end up to be considerably larger than 3.

In principle, the ‘conical’ surface could be described by an axial coordinate (encoding time-within-trial) and an angular one (encoding response angle). Thus, we would expect *ID* = 2. The best estimate by lFCI was *ID*_*lFCI*_ = 2.35 (C.I. [2.09, 3.89]). A closer inspection through the multiscale ID plot (in 3G) shows that local ID estimates are low for sufficiently small neighborhoods (K≲1000), while they become much higher for larger neighborhoods, eventually reaching values in the range [10, 20]. Yet, estimates obtained for K>rsim500 are discarded due to curvature and/or bad GoF. As a result, the local ID histogram has a clear peak around 2 (Fig 3H). The intrinsically 2-dimensional geometry of the manifold can be effectively captured using locally linear embedding (Methods), which yields an explicit 2-dimensional parametrization of the manifold. As shown in Fig 3I, the manifold can be ‘flattened’ on a two dimensional surface parametrized by a radial (time) and an angular (response angle) coordinate. However, the classical maximum likelihood ID estimator yields *ID*_*MLE*_ = 3.34 - considerably lower than parallel analysis, but definitely larger than 2. The Two-NN estimator gives *ID*_*TwoNN*_ = 7.03. As is well known, the maximum-likelihood estimator can be affected by the non-uniformity of the distribution, in this case probably causing an overestimate. The Two-NN estimate, in turn, is strongly biased by small-scale noise [47]. Only by reducing the density of the dataset through decimation (which reduces the effects of small-scale noise increasing the average distance between points), we obtained an estimate *ID*_*TwoNN*_ = 2.05 much more consistent with the lFCI one (S3 Text and S4 Fig).

In summary, even in this simple task, the neural manifold is highly curved and tilted in several dimensions. As a result, linear ID estimation methods considerably overestimate the dimension. On the contrary, lFCI yields an estimate that is congruent with the task structure and the possibility of parametrizing the manifold with two coordinates.

**Fig 4:**
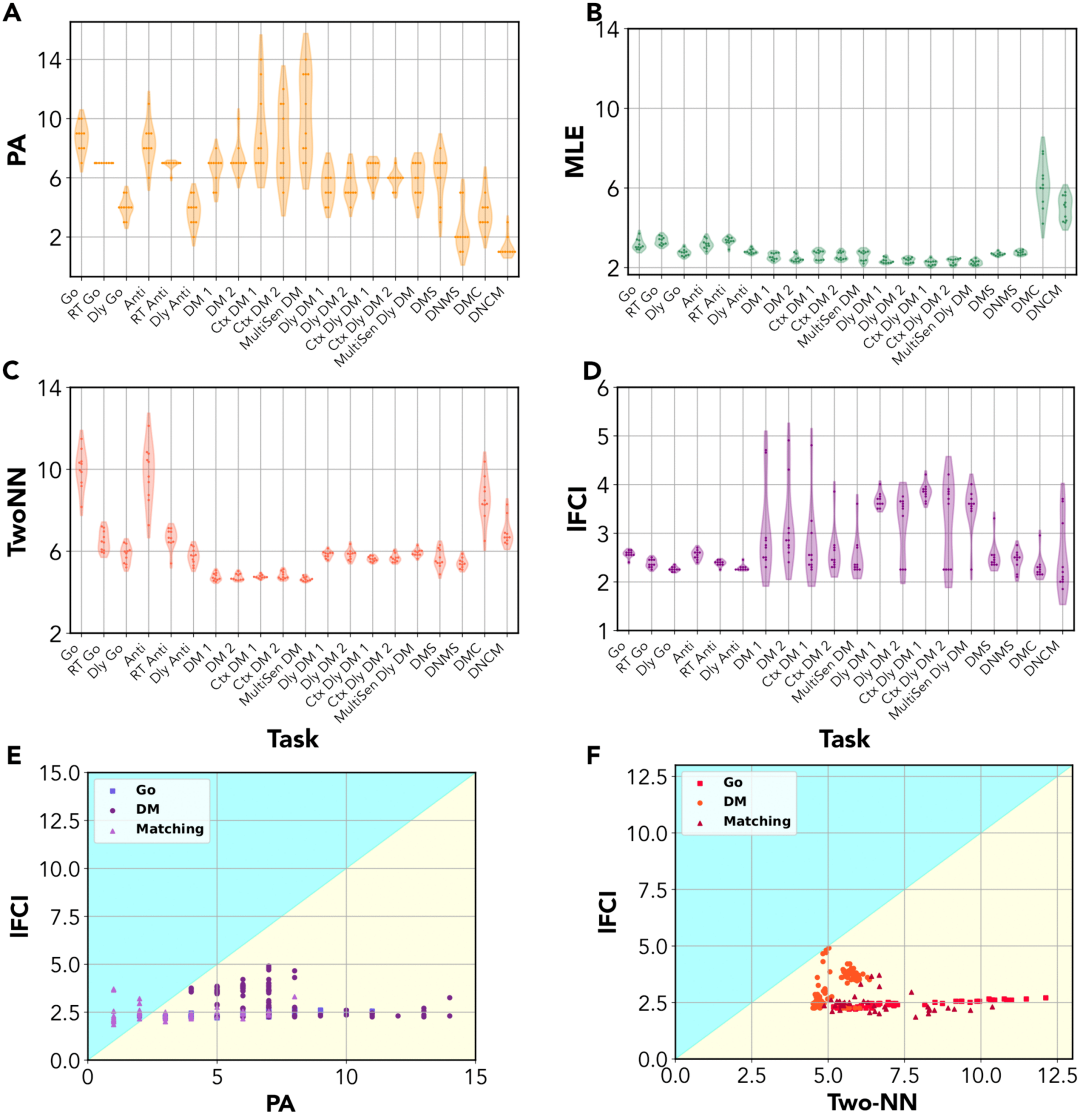
Intrinsic dimension of neural manifolds for the Cog-Task battery. For each task in the Cog-Tasks battery, we trained 10 independent recurrent neural networks (RNNs) and computed their ID with different methods. Notably, we compare estimates obtained by the local FCI (lFCI) method with those obtained with a linear ID estimation method, parallel analysis (PA) and two commonly used geometric ID methods, the classical maximum likelihood estimator (MLE) and the two-nearest-neighbors (Two-NN) estimator. **(A)** ID as the number of significant PCs identified with the parallel analysis (PA). For most tasks, ID estimates are not consistent in different trainings. Most estimates give $ID_{PA}\geq 5$. **(B)** ID estimated by the classical maximum likelihood estimator (MLE). MLE gives very consistent estimates, with $2\leq ID_{MLE}\leq 3$ for most tasks. The only exception is given by the delayed-match-to-category (DMC) task, which, however, is very difficult to train, with most networks learning the task only approximately. **(C)** For tasks in the ‘Go-Anti’ family, Two-NN gives quite inconsistent estimates, with $ID_{TwoNN}\geq 6$. For tasks in the ‘Decision Making (DM)’ family, estimates are consistent, with $ID_{TwoNN}\approx 5$ for the non-delayed and $ID_{TwoNN}\approx 6$ for the delayed tasks. For tasks in the ‘MS’ family, we obtain $ID_{TwoNN}\approx 5$ for D(N)MS, and $ID_{TwoNN}\geq 6$ for D(N)MC. **(D)** The local FCI (lFCI) method gives consistent estimates around 2.5 for all tasks in the ‘Go’ family, and estimates between 3 and 4 for tasks in the DM and MS family. **(E)** The ID identified by lFCI is always considerably lower than the ID estimate obtained by PA, except for the MS family. This is a reflection of the fact that neural manifolds for the Cog-Tasks battery are generally curved and extended across many linear dimensions, despite having an intrinsic 2- or 3-dimensional geometry. **(F)** The ID identified by FCI is always lower than the estimate obtained by Two-NN, which is severely affected by small-scale noise.

In Fig 4, we show ID estimates obtained when training 10 RNNs to solve each of the tasks. As for lFCI, we show the best estimate. For all tasks in the Go Family, we consistently obtain $2.2\leq ID_{lFCI}\leq 2.6$ (Fig 4D). The classical maximum-likelihood estimator gave ID estimates between 3 and 4 (Fig 4B). Two-NN gave larger and quite variable ID estimates, $ID_{TwoNN}\in [5,12]$ (Fig 4C). When applying decimation, which removes small-scale noise, we obtained estimates consistent with those of lFCI, i.e., around 2.5 (S3 Text and S4D and S4F Fig). This result is consistent with the above analysis of the react-Go task, showing that the manifold is essentially spanned by a circular and a temporal variable. Linear ID estimates by parallel analysis are in general much larger (up to *ID*_*PA*_ = 10) and strongly depend on the task and training instance ($ID_{PA}\in [6,11]$ for Go and Anti; $ID_{PA}\in [3,5]$ for Dly-Go and Dly-Anti; *ID*_*PA*_ = 7 for RT-Go and RT-Anti). Parallel analysis counts all directions on which the manifold has a non-vanishing projection, which broadly corresponds to the number of principal components explaining 90% of the variance (S4A Fig). Another linear ID estimation method, the participation ratio, effectively counts only the most relevant projections. The participation ratio yields indeed lower estimates than parallel analysis (in the range [1, 5], but estimates again strongly depend on the task and training instance (S3 Text and S4B Fig).

For the React-Go and React-Anti tasks, the participation ratio can yield estimates close to 1, a direct consequence of the presence of a ‘dominant’ principal component explaining a large fraction of the total variance (the cone ‘axis’ representing time-within-trial).

For tasks in the DM family, most trainings gave $2\leq ID_{lFCI}\leq 3$ for the non-delayed tasks (with a few outliers in the range [4, 5]), and $3.5\leq ID_{lFCI}\leq 4$ for the delayed tasks (with a few outliers around 2). We can trace back this increased dimension to a perturbation of the previously analyzed ‘conical’ manifold structure. Like in the Go family, neural trajectories must encode the response angle in the ‘Go’ phase to produce a correct response. However, in the delayed tasks (where the two stimuli are given in succession) the network must also transiently encode the first stimulus angle, which must be kept in memory until the second stimulus is presented and the correct response can be computed. Trajectories converging to the same attractor points thus form a smeared bundle. This bundle also includes ‘backward’ trajectories: as the stimuli are switched off in the ‘Go’ phase, trajectories do not remain in the vicinity of attractor points but slowly converge back to the origin before the end of the trial (for a detailed example and discussion, see S2 Text and S3 Fig). This ‘smearing’ of trajectories associated to the same attractor determines an additional ‘transversal’ degree of freedom, causing a rise in dimension. This phenomenon also occurs in the non-delayed tasks, where ‘smearing’ is only due to backwards trajectories not coinciding with the forward ones, causing only a minor dimension increase with respect to the Go tasks (~+0.5). ID Estimates obtained with classical *MLE* are consistent and slightly above 2 for all DM tasks (Fig 4B), suggesting that MLE identifies only the two local dimensions with the strongest variability. ID Estimates obtained with Two-NN are consistently larger than those of lFCI ($ID_{Two-NN}≃5$ for the non-delayed DM tasks, $ID_{Two-NN}≃6$ for the delayed ones; Fig 4C). Upon decimation, estimates are in good agreement with those of lFCI (S3 Text and S4D Fig). On the other hand, linear ID estimates are again quite inconsistent over tasks and trainings: parallel analysis generally yields much larger estimates ($ID_{PA}\in [4,15]$; S4A Fig), while the participation ratio yields estimates in a similar range ($2\leq ID_{PR}\leq 5$). The participation ratio estimates are not correlated with those of lFCI (S3 Text and S4E Fig), suggesting that the two methods capture different geometrical properties: *ID*_*PR*_ captures global variation along different orthogonal axes, and *ID*_*lFCI*_ captures local variation.

Finally, for tasks of the MS family, we consistently obtain $ID_{lFCI}\sim 2.5$ for DMS and DNMS. This result is fully consistent with estimates given by maximum-likelihood estimator and Two-NN (after decimation). Parallel analysis gives inconsistent and high estimates, while the participation ratio gives estimates close to 1, due to a dominant PC. For the DMC and DNCM tasks, most trainings give $2\leq ID_{lFCI}\sim 2.5$ (except one outlier around 3 for DMC, one outlier slightly below 2 and two outliers between 3 and 4 for DNCM). ID Estimates for DMC are thus more consistent than those of other estimators, while those for DNCM show some variability, which also emerges in maximum-likelihood and Two-NN estimates - perhaps reflecting a difficulty of properly training this task (the accuracy was 0.90±0.05 compared to 0.97±0.01).

### ID of synthetic neural recordings

Altan et al. [31] introduced a benchmark data set of synthetic neural recordings. As detailed in Methods, the data were generated by considering firing rate data from *d* independent recording channels, with varying d∈[3,6,10,20,40], embedding them in *N* = 96 dimensions with a linear transformation (*linearly embedded data*), and finally applying a non-linear transformation (*nonlinearly embedded data*).

**Fig 5:**
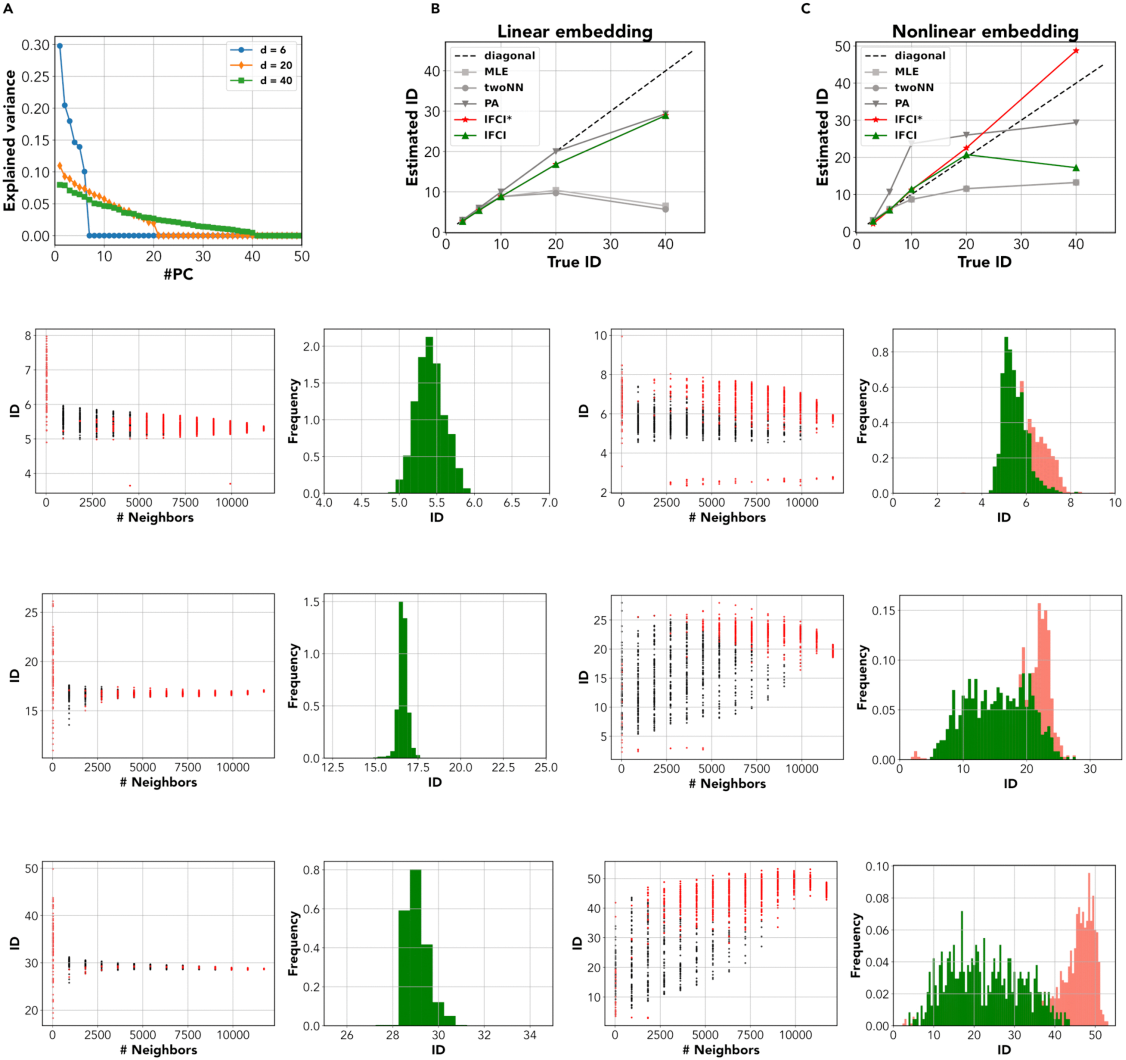
ID estimation for the synthetic neural data with increasing dimension. In Ref  [31], neural manifolds were constructed by randomly sampling firing rates from *d* independent channels in a multielectrode array. Data were then linearly embedded in 96 dimensions (*linearly embedded data*), and further subjected to a non-linear transformation (*nonlinearly embedded data*). **A** Variance explained by the principal components (PCs) of the linearly embedded data. **B** The ID was estimated by different methods, including parallel analysis (PA), Maximum Likelihood Estimator (MLE), Two-Nearest Neighbors (Two-NN), and the local FCI method; in red the lFCI estimates without our procedure to filter out the unreliable estimates, in green the lFCI results after the refinement. Dashed line indicates the identity. The linearly embedded data are stretched along *d* orthogonal axes, but the variance is very small ($≲10^{-2}$) for several components. All ID estimation methods yield ID~d for d≤10. For d≥10, MLE and Two-NN show a non-monotonic behavior, with ID≤10. PA and lFCI give monotonically increasing estimates for increasing *d*, with $ID_{lFCI}\sim ID_{TwoNN}\sim 30$; the presence of the unreliable results does not affect the final lFCI estimate. **C** The nonlinearly embedded data give rise to strongly non-smooth manifolds. All ID estimation methods yield ID~d for d≤10. For d≥10, MLE and Two-NN show a non-monotonic behavior, with ID≤10. lFCI shows a monotonically increasing trend, but the ID remains much lower than *d* ($ID_{FCI}\leq 13$). Keeping the unreliable estimates lFCI gives $ID_{lFCI}\sim d$ for all *d*. **D** For the linearly embedded data at *d* = 6, local ID estimates are consistent at all *K*, with a slightly decreasing trend for increasing *K*, and we obtain *lFCI* = 5.51 [5.23, 5.71]. **E** For the nonlinearly embedded data at *d* = 6, local ID estimates are consistent at all *K*, and we obtain *lFCI* = 5.51 [4.98, 6.25]. **F** For the linearly embedded data at *d* = 20, most local ID estimates are consistently in the range [15, 20] at all *K*, with a decreasing trend for increasing ***K*.** We obtain *lFCI* = 16.72 [16.45, 17.06], consistent with the number of PC components with variance >10^-2^. **G** For the nonlinearly embedded data at *d* = 20, local ID estimates are very variable (in the range [5, 20] for all *K* < 10^4^. Only for $K\approx 10^{4}$ do estimates become more consistent with values around 20. **H** For the linearly embedded data at *d* = 40, most local ID estimates are consistently in the range [23, 34] at all *K*, with a decreasing trend for increasing ***K*.** We obtain *lFCI* = 29.33 [28.77, 29.97], consistent with the number of PC components with variance >10^-2^. **I** For the nonlinearly embedded data at *d* = 40, local ID estimates are very variable (in the range [5, 40] for all *K* < 10^4^. Only for $K\approx 10^{4}$ do estimates become more consistent with values around 40.

The linearly embedded data present a challenge for classical geometric ID estimators, but not linear ones. While parallel analysis always finds ID≃d, geometric estimators find ID~10 for all d≥10, a reflection of their (known) difficulty in estimating high dimensionalities. Here, lFCI outperforms geometric methods, giving estimates broadly in line with those of parallel analysis: *ID*_*lFCI*_ =[3.00, 5.63, 8.87, 16.74, 29.41] for *d* = [3, 6, 10, 20, 40] (Fig 5B). In fact, this data does not pose significant challenges to lFCI, as reflected in the very well-behaved appearance of the multiscale ID plots and the local ID histograms for *d* = [6, 20, 40] (Fig 5D, 5F and 5H). Estimates are reliable (a natural consequence of the non-curved nature of the data) and consistent for all scales *K*, so that the local ID histogram is sharply peaked. The ID underestimation (which becomes evident for *d* = 20 and even more so for *d* = 40) is due to the presence of many dimensions with extremely small variance,  <10^-2^ (Fig 5A). In this condition, it is essentially impossible to reliably discriminate these dimensions from small-scale noise. In fact, also parallel analysis tends to identify a number of dimensions lower than *d*, and *ID*_*lFCI*_ and *ID*_*PA*_ converge for *d* = 40. To validate this hypothesis, we variance-normalized the data before the embedding. For these equal-variance data, we obtained $ID_{lFCI}≃d$ for all *d* (S6 Fig).

The nonlinearly embedded data have some very peculiar characteristics, which we summarize here (only some were discussed in [31]): i) the data distribution along each dimension is nearly log-normal. ii) The upper tails of the distributions give rise to isolated low-density regions along orthogonal axes (S5A Fig). In these regions, the ‘tangent plane’ has not a dimension equal to the ID, and perhaps is not even well-defined. iii) the density varies dramatically across the dataset (to measure this quantitatively, we used the inverse of the distance of the 10-th neighbor as a proxy of the density). As a result, the manifold contains a highly dense ‘core’ and a large, rarefied ‘periphery’ (this is visualized in S5B and S5D Fig, where we projected the manifolds for *d* = 20,*d* = 40 in two dimensions using t-stochastic neighbor embedding [73], showing the local point density).

Due to these joint characteristics, ID estimation becomes a difficult endeavor. Due to i), the covariance structure of the data is strongly influenced by outliers: this makes linear ID estimation unreliable. In fact, parallel analysis first overestimates (for *d* < 20), then underestimates (for *d* = 40) the ID. Geometric estimators are bound to underestimate the ID (for *d* > 10) due to their intrinsic limitations but also the manifold geometry, as nearest-neighbor statistics in the peripheral regions strongly deviates from the predicted distribution. Indeed, the maximum-likelihood estimator and Two-NN give $ID_{MLE},ID_{Two-NN}<d$ for d≥10. In this situation, lFCI can give accurate estimates up to *d* = 20, while it fails for *d* = 40, producing a sharp underestimation: *ID*_*lFCI*_=[2.8, 5.8, 11.3, 20.7, 17.2] for *d* = [3, 6, 10, 20, 40]. The problem for *d* = 40 can be mitigated by relaxing a step in the pipeline, the removal of estimates corresponding to bad GoF, although this comes at the price of some overestimation: *ID*_*lFCI*_=[2.5, 5.8, 11.2, 22.5, 48.7] for *d* = [3, 6, 10, 20 40]. To better analyze the reasons for this (partial) success, in Fig 5E, 5G and 5I we show the multiscale ID plots and the local ID histograms for *d* = 6,20,40. For small *d* (*d* = 6), the behavior is similar to the one observed in the linear embedding case. Although local ID estimates are slightly more variable (in the range [5, 7]), the local ID histogram is still sharply peaked For *d* = 20,40 local ID estimates appear to be highly variable for most neighborhood sizes *K*, except K≥10000. This behavior depends on the dataset geometry: local ID estimates strongly correlate with the density (S5C and S5E Fig). Neighborhoods in the highly dense ‘core’ yield local ID estimates ~d, whereas neighborhoods in the rarefied periphery cannot detect the full dimensionality, systematically yielding lower ID estimates. For large *K* (>rsim5000), all neighborhoods encompass a large fraction of the whole manifold. ID estimates become accordingly more consistent. However, estimates also become unreliable. This is not due to curvature (we always find δ~1 in our data, reflecting the absence of curvature), but rather to a bad GoF. In fact, large neighborhood must include regions with variable density, leading to a violation of the isotropy assumption and hence a deviation of the FCI curve from its theoretical shape. The FCI fitting procedure, however, spots a reasonable if slightly overestimated dimension. For *d* = 20, the local ID histogram has a peak around 20 even if unreliable estimates are discarded, even though the peak is shifted to ~22 and becomes more pronounced if they are included (5G). For *d* = 40, the local ID histogram has a peak around 20 unless unreliable estimates are included (5G), which produces a peak around 45. In synthesis: when considering small neighborhoods, the presence of rarefied regions tends to produce ID underestimation; when using large neighborhoods, this problem is solved but density variations imply a bad GoF and some overestimation. Nevertheless, even for this very challenging manifold, lFCI can yield rich insight into the manifold’s structure and ID.

### ID of high-dimensional visual responses

Having extensively benchmarked our method on artificial data in preceding sections, we now analyze real data from 2-photon imaging data of mice exposed to visual stimuli. Data were first discussed in the classical work by Stringer et al. [22,74] and made publicly available [67]. This data set includes responses of ~ 10,000 primary visual cortex neurons to several stimuli, including: 2,800 natural images, 32 natural images, 32 simple grating stimuli, and degraded natural images. Importantly, each visual stimulus was presented at least twice, allowing for a better discrimination of stimulus-related and stimulus-unrelated variability in neural recordings. Ref. [22] characterized the linear ID of neural responses. The ID found was always in line with the size of the stimulus set: for the small stimulus sets (32 images or gratings) it remained of the order of ≈30, while for the very high-dimensional stimulus (2800 images), it reached values of the order of $\sim 10^{3}$. Importantly, Ref  [22] provided solid evidence that this large linear dimension truly represents stimulus-related variability. Leveraging the availability of (at least) two repetitions of the same stimulus, they computed the cross covariance of responses across repetitions, which is provably a good estimate of stimulus-related variance. They then characterized relevant (linear) dimensions as axes with high cross-covariance (a method called cross-validated PCA or cvPCA, S4 Text).

While Stringer et al.’s findings suggest that visual responses are *not* organized in a low-dimensional hidden manifold, this conclusion cannot be drawn without an analysis of the ID. In fact, a large linear dimension may emerge from a high-ID geometry, but also from a low-ID, highly nonlinear geometry. Note that the latter case is not merely a mathematical curiosity, as some common neural codes were shown to produce exactly this type of highly non-linear manifolds [42], and a recent study showed that a high linear dimension can emerge in recurrent neural networks with a low-dimensional latent dynamics [44]. To adjudicate between these two scenarios, we re-analyzed Stringer et al.’s data using lFCI alongside other ID estimators. We focused our analysis on data from a mouse for whom responses to multiple stimulus sets were recorded. To remove stimulus-unrelated variability, we adopted the same logic as Ref  [22], and we first projected the data onto a (linear) subspace containing 99% of stimulus-related variance, as estimated by cvPCA. While this step effectively removes stimulus-unrelated dimensions, it maintains the non-linear geometry of the stimulus-related responses - if any (projecting a nonlinear manifold onto a hyperplane yields a linear manifold only if all the nonlinearity is contained in the dimensions orthogonal to the hyperplane).

**Fig 6:**
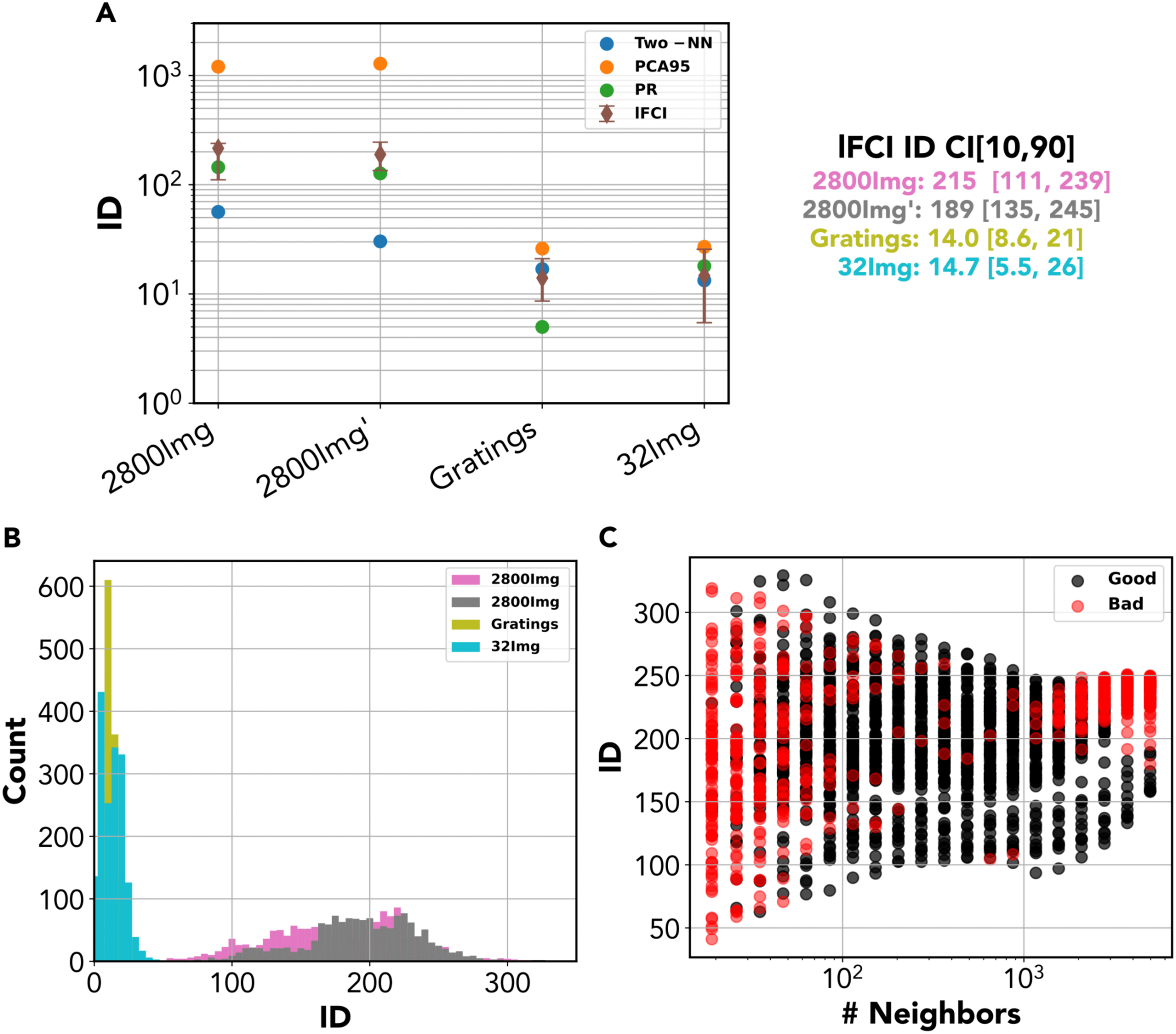
ID of High-dimensional visual responses. Results of the ID analysis on the mouse visual responses. The data have been processed with cvPCA before the dimensionality analysis. **A** ID results for the different experiments; **B** Distribution of the lFCI ID values for the different experiments; **C** ID values for the different neighborhood sizes for the experiment with 2800 images. The analysis for the curvature shows that the dataset is flat with δ<1 for all the experiments.

In the two experiments with 2,800 images, the linear dimension (as estimated via PCA with α=0.95) is very large, ~1,200 (Fig 6A). This is due to many of the linear dimensions having a very low variance: the effective linear dimension (as estimated by the participation ratio) is nearly one order of magnitude lower, around ~140. We estimated the ID using Two-NN and lFCI. Two-NN yields inconsistent estimates in the two replications - respectively, ~55 and ~30. While lFCI estimates display a relatively large interval for both repetitions ($ID_{lFCI}\in [115,245]$, $ID_{lFCI}\in [111,239]$), the local ID histogram is peaked (Fig 6C) and the best estimates broadly in line with those of the participation ratio, $ID_{lFCI}=215,ID_{lFCI}=189$. To properly interpret this discrepancy, one should take into account that Two-NN tends to systematically underestimate the ID when above ~20, and radically so for ID>rsim100 (S1 Fig) Although it would be tempting to explain the difference between Two-NN estimates and linear dimension estimates in terms of curvature, this explanation is not supported by the data. In fact, lFCI failed to detect significant curvature in the data: the distribution of δ was entirely contained in the range [0,1] for all neighborhood sizes (the 95% percentile of the δ distribution was 0.65 in the first replication and 0.65 in the second). The comparatively low estimates given by Two-NN are then probably attributable to a method’s bias.

In the experiment with a reduced stimulus set of 32 natural images, both the linear dimension and the ID dropped dramatically with respect to what seen for the full stimulus set. PCA yielded a value ~28, roughly equivalent to the maximum dimension (equal to the size of the stimulus set). Estimates by the other methods were broadly consistent: the participation ratio yields ~171, Two-NN gives $ID_{Two-NN}\sim 13$, lFCI $ID_{lFCI}\sim 20$. Despite the decrease in dimension, responses do not seem to be arranged in a truly low-dimensional manifold. In the experiment with gratings, results were qualitatively similar, except that we observed a further decrease of the effective linear dimension estimated by the participation ratio, which is ~5. The linear dimension estimated with PCA is ~27, close to the maximum possible value. Two-NN gave $ID_{Two-NN}\sim 17$, $ID_{lFCI}\sim 12$.

In summary, our analysis suggests that visual responses to a high-dimensional set of natural images are organized within a high-dimensional, linear manifold with dimension ~150. Even when considering a much smaller stimulus set, or radically simpler stimuli such as grating, there is no evidence of a truly low-dimensional organization. We remark that these conclusions remain valid if one does not preliminarily remove stimulus-unrelated variance (S4 Text and S7 Fig): the main differences are that (i) for the stimulus sets with 2,800 images, the picture is similar but stimulus-unrelated noise leads to even larger dimension estimates (ii) stimulus-unrelated variability outbalances stimulus-related one in the case of the stimulus sets with 32 stimuli, leading to much larger dimension estimates.

## Discussion

The dimensionality of neural population activity is at the root of key open issues in computational neuroscience, such as structural constraints on the geometry of neural population activity [18,20,75], the relation between spontaneous and task-evoked activity [22,74,76], the development of abstract codes [24,26,77], and the number of recorded neurons needed to exhaustively characterize neural population responses. More generally, characterizing the dimensionality of neural manifolds is critical to understand how neuronal populations collectively encode relevant variables and perform computation (see classic [7] and recent [10] reviews on neural manifolds). The ongoing debate could be affected by the limitation of common methodologies to measure the ID of neural manifolds [17]. Linear methods (PCA and its evolutions) are inappropriate in the case of strongly nonlinear manifolds, while geometric ID estimators (which have been often employed to study representations of deep artificial neural networks [78–81]) can dramatically underestimate the ID in the case of high dimensionality.

### Methodology

Here, we introduced a novel ID estimation pipeline based on a local version of the full-correlation integral (Fig 1). Our pipeline, called local FCI or lFCI, computes the ID locally on neighborhoods of variable size, obtaining a robust ID estimate from the histogram of local estimates (Fig 2). Importantly, we prune the histogram from unreliable local estimates obtained on neighborhoods with significant curvature or density variations. As shown by the summary of results in Table 1, our pipeline is versatile enough to reliably cover a wide range of dimensionalities and non-linearities. In all cases analyzed, lFCI performed comparably or better than other methods. Importantly, no other method (among those included in our analysis) was able to produce consistently reliable results in a wide range of dimensionalities. Our pipeline encountered tangible difficulties only in the extreme case of a high-dimensional, highly skewed data distribution; even in this case, relaxing the pipeline yielded correct insight on the ID (Fig 5). Table 2 shows the lFCI results with the confidence interval. It is worth noticing that the True ID is included in the confidence interval. In the linear embedding case, the ID is underestimated due to the presence of some dimensions that have a very small variance.

**Table 1:** Summary of the results.

Task name	True ID	PA	MLE	twoNN	lFCI
cog-task (GO)	~2	6.43	3.09	7.42	**2.41**
cog-task (DM)	~3	7.08	2.23	5.26	**3.16**
cog-task (Match)	~3	3.40	4.14	6.63	**2.45**
neural traj. (lin)	3	3.00	3.04	2.99	**2.70**
	6	6.00	5.94	5.88	**5.51**
	10	10.00	8.82	8.77	**8.81**
	20	20.00	9.70	10.40	**16.72**
	40	29.33	5.66	6.54	**29.33**
neural traj. (exp)	3	3.00	2.98	3.02	2.70 (2.13*)
	6	10.67	6.10	6.04	5.51 (5.77*)
	10	23.67	8.65	8.69	9.31 (11.20*)
	20	26.00	11.58	11.52	16.22 (22.51*)
	40	29.33	13.20	13.24	17.42 (48.72*)

**Table 2:** Summary of the results for lFCI.

Task name	True ID	lFCI C.I. [10,90]
neural traj. (lin)	3	**2.70 [2.62, 2.96]**
	6	**5.51 [5.23, 5.71]**
	10	**8.81 [8.55, 9.08]**
	20	**16.72 [16.45, 17.06]**
	40	**29.33 [28.77, 29.97]**
neural traj. (exp)	3	**2.70 [2.05, 3.22]**
	6	**5.51 [4.98, 6.25]**
	10	**9.31 [6.94, 11.39]**
	20	**16.22 [8.92, 21.87]**
	40	**17.42 [11.85, 39.73]**

In future developments, we might integrate this pipeline with suitable denoising procedures to further enhance its robustness. In Ref. [31], Altan et al. proposed the following denoising/estimation scheme: 1) use parallel analysis (PA) to obtain an upper bound on the ID, *D*_*u*_; 2) denoise the data by first projecting (linearly, via PCA, or non-linearly, via an autoencoder) onto a *D*_*u*_-dimensional space, and then projecting back into the original space; 3) if nonlinear denoising is more effective than linear denoising (indicating a nonlinear manifold) use a geometric ID estimator; otherwise, use a linear one. Our results show that FCI can yield a more robust upper bound than PA, also valid in a highly nonlinear case. We thus suggest the following modification of Altan et al.’s scheme: 1) use FCI to obtain an upper bound on the ID, *D*_*u*_; 2) denoise the data 3) compute a handful of local ID estimates for small neighborhoods (e.g., *M* = 10 centers and K∈[50,100,200]) and average the resulting estimates 4) if there is agreement with the global estimates (small relative error), stop; otherwise, perform to the full lFCI procedure to characterize the ID (note that step 3, which can be performed rapidly at a low computational cost, can spare the computational burden of the complete lFCI procedure when probably not needed).

#### ‘Embedding’ vs. ‘intrinsic’ dimension of neural manifolds.

In Ref  [41], Jazayeri et al. explicitly distinguished between the ID and the minimum dimension of a linear subspace containing the manifold, which they termed ‘embedding dimension’ (ED). Following their terminology, linear methods (such as parallel analysis, participation ratio, and PCA with a fixed variance threshold) essentially measure the ED. Due to their (undeniable) advantages, such as simplicity and robustness, many research works employ linear methods (see, e.g., [14,15,20,26,27,32–37,39]), and many recently proposed developments for dimensionality reduction are also based on linear projections [23,33,38,40]. However, since neural manifolds can be highly non-linear and curved [42], paying attention to the ID/ED distinction may be relevant in many cases. A paradigmatic example is provided by our analysis of neural manifolds of RNNs trained to perform the Cog-task battery (Figs 3 and 4) Albeit intrinsically low-dimensional, these manifolds had non-vanishing projections along several orthogonal axes, and the ED was considerably higher than the ID. Importantly, the ID was i) more clearly related to fundamental task variables such as response angle and integration time ii) much more stable than ED across trainings, suggesting that the ID could be among the ‘universal’ properties [82] that do not depend on network architecture and training, but only on the computational scaffold underlying the task [82]. In a classic paper, Gao et al. proposed an upper bound to the neural manifold dimension in terms of the ‘neuronal task complexity’ [36], an index depending on the autocorrelation of activity for similar values of task-relevant variables (such as response angle and integration time). In fact, the neuronal task complexity bound applies to a measure of ED, the participation ratio, and this bound is generally quite loose and much larger than the ID (this is shown in S4 Fig for the ‘Cog-Task’ battery).

#### Low-dimensional neural manifolds for cog-tasks.

Recent literature has abundantly mentioned ‘low-dimensional neural manifolds’. In experimental studies, the low-dimensionality was generally inferred from the fact that few principal components could explain a large fraction of variance (see, e.g., the seminal article by Churchland et al. demonstrating that a reaching task induces a low-dimensional neural activity [15]). However, only rarely was a rigorous ID characterization performed (an exception is [17]). In turn, theoretical studies have often investigated cases where neural activity was compelled to be low-dimensional by structural constraints, such as a low-rank connectivity [18,19,61]. To what extent networks *trained* to perform simple tasks would exhibit a low dimensional activity was therefore an open question. In this work, we have analyzed the activity of RNNs performing the common ‘Cog-Task’ battery, showing that their activity is indeed organized in low-dimensional (approximately 2- or 3-dimensional) manifolds (Fig 4). The low dimensionality seems to be directly related to the simplicity of the tasks at hand. The only key variable the networks must encode is the response angle, which can be encoded by a ‘ring’ of attractors (Fig 3). Additional dimensions come from the need to switch these representations on and off, departing or returning to a state of zero activity. The minimal increase in dimension is + 1, but it can be larger depending on the divergence of forward and backward trajectories. Future studies could investigate more complex tasks such as the ‘Mod-Cog’ battery proposed by [51].

#### High dimensional visual responses.

The article by Stringer et al. [22] contained a landmark finding, as it provided robust evidence that neural activity can be very high-dimensional. We reanalyzed the same data to test whether using a non-linear ID estimator would alter, or circumscribe, the main conclusions reached in that work. Our analysis based on lFCI offered substantial backing to the picture drawn by Stringer et al. by showing that visual responses to a large set of visual stimuli are organized in a linear, high-dimensional manifold - and not in a non-linear, low-dimensional one (Fig 6). While the ID is lower than the embedding dimension estimated by PCA, its value (~150) aligned with the effective linear dimension corresponding to the most relevant principal components, as estimated by the participation ratio. When analyzing responses to a small random subset of 32 stimuli (from the same set of 2,800 stimuli), the ID was considerably reduced (~15), but we found no evidence of truly low-dimensional activity. This is not entirely surprising, because the resulting manifold should emerge from neural configurations that are randomly sampled from the global high-dimensional manifold.

Overall, this analysis, coupled with the previous one on ‘Cog-Tasks’, support the hypothesis that a primary determinant of the neural manifolds’ ID is the complexity of the stimulus-space underlying the observed neural responses.

#### Limitations.

The main limitation of the lFCI method is that it assumes a smooth manifold structure. In the presence of non-smooth, sparsely or radically non-uniformly sampled manifolds, lFCI’s assumptions can be violated. Some of the dimensions can be poorly sampled locally, yielding local ID estimates that are significantly lower than the global ID of the manifold. This was partly evidenced in our analysis of nonlinear data from [31], Figs 5 and S5), where low-density, peripheral regions yielded a lower local ID than the high-density bulk of the manifold. As long as real recordings do not present similar strongly skewed, fat-tailed firing rate distributions, lFCI should perform reliably. Related to the first, a second limitation is lFCI’s dependence on the isotropy assumptions. While this assumption is often well-satisfied locally, it can be violated in the presence of strong density gradients, leading to a poor GoF and, possibly, biased estimates. While we decided to stick to a conservative criterion, whereby we discard local estimates with comparatively bad GoF within the same dataset, ideally one could try to gauge the GoF filter, and possibly correct biases, depending on the measured density gradient in the data. Future work may address this point through a wide array of numeric experiments.

Finally, in this work we did not discuss how to obtain low (or lower-) dimensional representations once a solid ID estimate is obtained (we only made a cursory example in the discussion of the neural manifold for the React-Go task). In principle, many approaches for nonlinear dimensionality reduction are available for this aim: from classical methods such as locally linear embedding to more modern ones such as deep autoencoders (see Methods for a brief review). Nearly all of these methods include the ID as a free parameter, so ID estimation would serve as a crucial preliminary step. Future work might address this issue more in depth.

#### Conclusion.

In conclusion, we proposed a robust pipeline to estimate the intrinsic dimension of neural manifolds, offering a new tool to tackle an open debate. The inherent properties of lFCI make it robust to undersampling and nonlinearity, allowing us to probe a large range of IDs. Our pipeline allowed us to rigorously demonstrate that simple tasks induce a low-dimensional structure in neural activity and highlight the difference between the ID and the ‘embedding dimension’ given by linear estimators.

## Supporting information

S1 Text Additional methodological remarks on lFCI. (PDF)

S2 Text Intrinsic dimension of neural manifolds for the Cog-Task battery - Neural manifold for Ctx-DM1. (PDF)

S3 Text Intrinsic dimension of neural manifolds for the Cog-Task battery - Additional Results. (PDF)

S4 Text Intrinsic dimension of high-dimensional visual responses - additional notes. (PDF)

S1 FigScaling of FCI and Two-NN estimates with increasing D.We show results of FCI on *M* uniformly sampled data ($M\;=\;10^{2},\;10^{3},\;10^{4}$) on hypercubes of known dimension varying from *D* = 10 to *D* = 1000. Results are compared with those of Two-NN, a common ID estimator.(TIF)

S2 Fig Examples of Goodness-of-Fit (GoF) for FCI. (TIF)

S3 Fig Dimensionality of the neural activity manifold in the Ctx-DM1 task. **A** Input, output and network activity for the Ctx-DM1 task; **B** Input, output and network activity for the Ctx-DM1 task; **C** Explained variance of the different PCs of the network activity. Looking at the variance, PCA predicts a dimensionality of 1 if we consider the highest drop in variance or a dimension of 7 if we consider the second elbow in variance. **D** Projection of the network activity on the first three PCs. The color code stands for time-within-trial (time from trial onset). *PC*_1_ is nearly aligned with time-within-trial. **E** Projection of the network activity on the first three PCs. The color code stands for response angle. **F** Multiscale ID plot for the RT-Go task. At low *K*, estimates are close to 3. **G** Local ID histogram, showing a peak at *D* = 3.26. **H** Locally linear embedding can flatten the manifold for the CTx-DM1 task onto a 2-D surface but only with some approximation.
(TIF)

S4 Fig Intrinsic dimension of neural manifolds for the Cog-Task battery. For each task in the Cog-Tasks battery, we trained 10 independent RNNs to solve the task and computed the ID of the neural activity manifolds for each case. **(A)** ID as the number of PCs identified with the principal component analysis (PCA) keeping the first principal components explaining 95% of the variance **(B)** ID estimated by the participation ratio (PR) **(C)** upper bound on the ID given by neuronal task complexity (NTC) **(D)** ID computed through the multiscale (with decimation) Two-NN method. **(E)** NTC can under- and overestimate the ID **(F)** The ID identified by the multiscale Two-NN method and the local FCI are in good agreement.
(TIF)

S5 Fig Local ID estimates and density for the ‘multielectrode array’ data. **(A)** TSNE embedding of the data with *d* = 20. Low local IDs are found in the ‘periphery’, while high local IDs are found in the ‘core’ **(B)** local IDs estimates strongly correlate with the local density of points for *d* = 20. **(C)** same as (A) for *d* = 40. **(D)** local IDs estimates strongly correlate with the local density of points for *d* = 20.
(TIF)

S6 Fig ID estimation for the ‘multielectrode array’ data with equal variance. (TIFF)

S7 Fig Analysis of the High-Dimensional Visual Responses. Results of the raw high dimensional visual responses recorded by Stringer [67]. **A** ID and ED values for the different experiments; **B** lFCI ID distribution for the different experiment. **C** ID as a function of the number of neighbors for the experiment with 2,800 images.
(TIF)

S8 FigThe distribution of curvature (δ) for flat manifolds.Distribution of the values of for three different dataset with increasing anisotropy and fixed *ID* = 3. **(A)**, α=0 corresponds to an isotropic distribution. **(B, C)**, α=1 and α=2 correspond to non isotropic distributions as points tend to accumulate around the origin and the axes. The data for α=2 are already strongly non-isotropic as they display a strong density gradient. For each data set, we considered local neighborhoods of varying size *K* and computed δ and GoF. In panels **D-F**, we show the resulting distributions of δ for *ID* = 3. The distributions are only mildly affected by α and the values remain strictly bounded. In particular, the 99-th percentile of the δ distribution is always lower than δ=2 (red dashed line in the plots).(TIFF)

S9 Fig The distribution of GoF for flat manifolds. **A-C** show the distributions of GoF, grouped according to neighborhood size *K*. In this case, the distributions are strongly affected by *α*; in red the distribution with the lowest median. For *α* = 0 the largest neighborhoods yield the most consistent GoF distribution. For *α* = 1, the distribution becomes clearly bimodal for all *K* ≥ 1000; For *α* = 2, the effects of anisotropy are so strong that most large neighborhoods (*K* ≥ 1000) yield *GoF* > 0.01. In this case, the neighborhoods yielding the most consistent GoF distribution correspond to *K* ≈ 150. **(D-F)** ID distributions for the different, in yellow the distribution for all the estimates, in green the distribution of ID after the filtering process. To ensure that analogous results could be obtained in a wide range of dimensions we repeated the same analysis for *D* = 3, *D* = 10, *D* = 20, *D* = 40. **(G)** the 99-th percentile of the distribution, that always remains below *δ* = 2. **(H)** the 99-th percentile of the GoF distribution corresponding to the *K* yielding the lowest median GoF. We see that the GoF threshold is highly variable from dataset to dataset. In all cases, removing ‘bad’ estimates improved the ID estimate **(I)**.
(TIF)

S10 Fig ID distribution as a function of the number of centers. For the Swiss Roll, we computed the local ID histogram varying the number of centers *M* = 10,30,100,300,1000. The number of centers affects only marginally the distribution of the ID estimates. The size of the neighborhoods is the parameter that most affects the estimation.
(TIF)
