# Supplementary material for: Exploring neural manifolds across a wide range of intrinsic dimensions
Source: PLoS Comput Biol. 2026 Apr 3;22(4):e1014162. doi: 10.1371/journal.pcbi.1014162 (PMC13068349; doi:10.1371/journal.pcbi.1014162)
Supplement: S1 Text — (PDF) [file pcbi.1014162.s001.pdf]

S1 Additional methodological remarks on lFCI

Computational cost of lFCI. We conclude this section by briefly discussing the computational cost of our method. The computational bottleneck of the procedure is the computation of pairwise distances, which needs to be performed for each local neighborhood. The calculation of pairwise distances scales as $\mathcal{O}(K^2D)$ for a neighborhood of size K and ambient dimension D. When using M centers and a logarithmic step in K (hence, $K = N/a^n$ for $a = 1, ..., N_K$, we obtain a total of

$$MD\sum_{n=0}^{N_K}(N/a^n)^2 = MDN^2\sum_{n=0}^{N_K}a^{-2n} = MDN^2\frac{1-a^{-2(N_K+1)}}{1-a^{-2}} = MDN^2g(N_K)$$

For reasonable choices of logarithmic step ($a > 1.3$) the function $g(N_K)$ quickly saturates and we have $g(N_K) < 4$. Hence, overall, we obtain a scaling of $\mathcal{O}(N^2DM)$. All other steps are less burdensome. For instance, K-neighborhoods are found by KD-a tree search algorithm, which scales as $\mathcal{O}(\log K) < \mathcal{O}\log N$. By using M centers and N_K neighborhood sizes, K-neighborhood retrieval has an overall cost of $\mathcal{O}(MN_K \log N)$.
As N and D are fixed by the data set, the major free parameter entering the computational cost is the number of centers, M. Using all points as centers is inefficient, since estimates will be largely redundant, especially for large neighborhoods that will end up having large intersections. For the small neighborhoods, ideally, one may try to include a number of neighborhoods covering the whole data set. When using $M = N/K$ randomly selected centers one expects to obtain an approximate covering including roughly 65% of all points (the probability of a point not falling in any neighborhood will be $(1 - K/N)^{N/K} \approx e^{-1}$ for $K/N \ll 1$). Thus, for a typical data set size of $N = 10000$ points, and a minimum K of 100, one obtains an approximate covering with $M = 100$ centers. Anyway, including a large number of centers will not significantly strengthen estimates. For the Swiss Roll, we computed the local ID histogram for $M = 10, 30, 100, 300, 1000$. Results are shown in S10 Fig, where we display the distribution of the ID as a function of M. It is apparent that estimates stabilize already at $M \sim 100$ and that the relevant parameter is the neighborhood size K, not the number of centers.
For our results, we used $M = 100$ centers, which ensures an approximate covering of the whole dataset even when using small neighborhoods ($K \sim 100$).

Automatic pruning of the local ID histogram. In lFCI, the size K of the local neighborhoods from which ID estimates are obtained is not fixed - in fact, local estimates at various K are a priori included. Local ID estimates are pruned away if either i) neighborhoods are provably non-flat, strongly departing from the local tangent plane, as signified by a large barycenter-to-neighborhood distance (δ), or ii) the quality of the fit degrades, as signified by a worse GoF.
The curvature thresholding is a core feature of our method: in its absence, we would systematically overestimate the ID in the case of curved manifolds. A low curvature ($\delta \sim 1$) is a robust feature of data living on flat manifolds. Extensive tests (details in the next paragraph) showed that when data are sampled on flat manifolds, δ remains in the range $[0, 2]$ with virtual certainty. Therefore $\delta > 2$ is a robust and conservative criterion for curvature detection, which justifies restricting attention to neighborhoods with $\delta < 2$.
The GoF thresholding is not critical, but it can contribute to 'polishing' the ID histogram, increasing estimation accuracy. The range of GoF can widely vary from dataset to dataset, depending on whether the isotropy condition is well respected. Therefore, we cannot discard local estimates on the basis of a unique, dataset-independent GoF threshold. Instead, we prefer to select a dataset-dependent threshold based on the

GoF distribution of the specific dataset at hand. When some of FCI's assumptions are violated, the distribution is shifted to worse (i.e., larger) GoF values and it is much broader or even multimodal (details in the next paragraph). In fact, neighborhoods at larger scales K can yield much worse GoF than others - because they are more curved, or because the isotropy violations become more prominent. We then first identify the scale (K) with the lowest median GoF, and by considering neighborhoods of size K we obtain a 'reference' GoF distribution. Estimates with GoF values that are outliers with respect to this distribution are considered 'bad' and discarded.
In summary: we discard local ID estimates based on i) a robust, universal curvature detection criterion or ii) GoF values that are exceedingly bad compared to other local estimates from the same dataset.

Distributions of delta and GoF on hyperplanes. We now provide support for our pruning procedure by analyzing data sampled from flat manifolds (hyperplanes). To explicitly account for possible effects of isotropy violations, we considered both isotropic and non-isotropic distributions. To this aim, we sampled $N = 10000$ points uniformly from D-dimensional hypercubes $[0, 1]^D$. We implemented the nonlinear transformation

$$x \to \frac{e^{\alpha x} - 1}{e^{\alpha} - 1}$$

with $\alpha = 0, 1, 2$. The case $\alpha = 0$ corresponds to an isotropic distribution. The cases of $\alpha = 1$ and $\alpha = 2$ correspond to non-isotropic distributions as points tend to accumulate around the origin and the axes. Data for $D = 3$ are shown in Fig S8 A-C. The data for $\alpha = 2$ are already strongly non-isotropic as they display a strong density gradient. For each data set, we considered local neighborhoods of varying size K and computed δ and GoF.
In Fig S8 D-F, we show the resulting distributions of δ for D=3. The distributions are only mildly affected by α and the values remain strictly bounded. In particular, the 99th percentile of the δ distribution is always lower than $\delta = 2$. The same holds true for other values of D: in Fig S9 G, we display the 99th percentile of the δ distribution for $D = 10$, $D = 20$, $D = 40$, which remains below $\delta = 2$. This shows that for isotropic or non-isotropic distributions on flat manifolds, δ remains almost certainly within the range $[0, 2]$.
In Fig S9 A-C we show the distributions of GoF, grouped according to neighborhood size K. In this case, the distributions are strongly affected by α. For $\alpha = 0$ the largest neighborhoods yield the most consistent GoF distribution. We identified the GoF distribution with the lowest median ($K = 7,300$; median GoF = 0.002) and computed its 99th percentile obtaining GoF = 0.0034. Local ID estimates with a larger GoF are considered 'bad'. When removing these estimates from the peak, we improved ID estimation (Fig. S9 D), from 2.94 [2.78, 3.17] to 2.93 [2.82, 3.02]. For $\alpha = 1$, the distribution becomes clearly bimodal for all $K \gtrsim 1000$, with a lower mode around GoF=0.002 and a higher mode around GoF=0.015. Thus, for $K \gtrsim 1000$ a sizable fraction of the neighborhoods can yield consistently bad GoF. In fact, while the point distribution is approximately uniform close to the origin, the effects of anisotropy became pronounced for neighborhoods centered far from the origin. Here, the neighborhood yielding the most consistent GoF distribution corresponds to $K \approx 200$. We determined the 99th percentile of the GoF distribution obtaining GoF=0.008. Local ID estimates with a larger GoF are considered 'bad'. When removing these estimates from the peak, we again considerably improved ID estimation (Fig S9 E), from 2.91 [2.65, 3.15] to 2.94 [2.78, 3.06]. For $\alpha = 2$, the effects of anisotropy are so strong that most large neighborhoods ($K \gtrsim 1000$) yield GoF > 0.01. In this case, the neighborhoods yielding the most consistent GoF distribution correspond to $K \approx 150$. We determined the

99th percentile of the corresponding GoF distribution obtaining GoF=0.01. Local ID estimates with a larger GoF are considered 'bad'. When removing these estimates from the peak, we considerably improved ID estimation (Fig S9 F), from 2.81 [2.59, 3.15] to 2.91 [2.63, 3.10].

To ensure that analogous results would be obtained in a wide range of dimensions we repeated the same analysis for $D = 3$, $D = 10$, $D = 20$, $D = 40$. In Fig S9 H, we display the 99th percentile of the GoF distribution corresponding to the K yielding the lowest median GoF. We see that the GoF threshold is highly variable from dataset to dataset. In all cases, removing 'bad' estimates improved the ID estimate (Fig S9 I).
