# Supplementary material for: Exploring neural manifolds across a wide range of intrinsic dimensions
Source: PLoS Comput Biol. 2026 Apr 3;22(4):e1014162. doi: 10.1371/journal.pcbi.1014162 (PMC13068349; doi:10.1371/journal.pcbi.1014162)
Supplement: S2 Text — (PDF) [file pcbi.1014162.s002.pdf]

S2 Intrinsic dimension of neural manifolds for the Cog-Task battery - Neural manifold for Ctx-DM1

In the Ctx-DM1 task, the network must ignore the stimuli presented in modality 2 ($\mathbf{u}_{mod2}$), and respond in the direction of the stronger of two stimuli presented in modality 1 ($\mathbf{u}_{mod1}$), as soon as the fixation cue goes off (S3A Fig). Thus, the task is conceptually very similar to Fd-Go, except that the response angle is a more complex function of the input (the network should also ignore any input in modality 2, but this requirement can be trivially satisfied by having weak input connections from inputs units corresponding to modality 2 and recurrent units). Another difference is that the stimuli are turned off as soon as the 'Go' signal starts, which, as we shall see, has a relevant effect on the structure of the neural manifold.

In Fig S3B we display snapshots of neural trajectories at different values of time-within-trial, coloring the points according to response angle. Before stimulus presentation, the RNN activity is at the origin ($\mathbf{r} = 0$). Stimulus presentation determines a weak displacement from the origin. After the fixation cue goes off, activity moves towards a set of attractors representing different response angles. Similar to the case of RT-Go, these attractors are arranged onto an approximately ring-like structure on which response angle is represented as a circular variable. However, the structure of this ring is much less regular than in the RT-Go case. Because input ceases as soon as the fixation cue goes off, after reaching this 'ring', trajectories slowly go back towards the origin. Importantly, the 'backward' trajectories are not fully equivalent to the 'forward' trajectories (S3D), creating transversal additional variation along a direction orthogonal to the ones encoding for angle and time-within-trial (conferring a sort of 'thickness' to the surface). When visualizing the entire manifold (S3E Fig), a highly twisted conical surface emerges. In Fig S3C, we plot the percentage of variance explained by several principal components. The first two components explain, respectively, 24% and 20% of the variance; the five subsequent components explain between 5% and 12% each. As a consequence, a linear ID estimation method, parallel analysis (PA) gives $ID_{PA} = 7$. We estimated the ID with the lFCI. The multiscale ID plot (S3F Fig) shows that local ID estimates for $K \geq 10^3$ are increasingly unreliable (worse GoF). Estimates for $K \leq 10^3$ are mostly in the range $[2, 5]$. The local ID histogram (S3G Fig) shows a broad peak between 3 and 4, giving $ID_{lFCI} = 3.26$. Consistently, MLE yields $ID_{MLE} = 3.34$. The Two-NN estimator gives a much larger estimate $ID_{Two-NN} = 7.03$, probably a consequence of small-scale noise. When using a multiscale version of Two-NN, we obtain indeed $ID_{Two-NN} = 3.78$.

We tried to project the manifold in two dimensions using locally linear embedding (S3H Fig). LLE can only approximately 'flatten' the manifold onto a two-dimensional ring where the radial coordinate represents time-within-trial, and the angular coordinate represents response angle. Yet, some of the response angles are badly represented, probably because of the inability of LLE to separate the backward and forward trajectories.
