# Supplementary material for: Exploring neural manifolds across a wide range of intrinsic dimensions
Source: PLoS Comput Biol. 2026 Apr 3;22(4):e1014162. doi: 10.1371/journal.pcbi.1014162 (PMC13068349; doi:10.1371/journal.pcbi.1014162)
Supplement: S3 Text — (PDF) [file pcbi.1014162.s003.pdf]

S3 Intrinsic dimension of neural manifolds for the Cog-Task battery - Additional Results

In Fig S4A, we show ID estimates obtained by counting the number of principal components (PCs) explaining 95% of the variance. These estimates are generally in line with estimates given by PA.

In Fig S4B, we show ID estimates obtained by the participation ratio (PR), which effectively counts only the most relevant PCs. For tasks in the Go family, PR yields lower estimates than PA (in the range $[1, 5]$, with estimates depending on the training instance. We obtain $ID_{PR} = 3.5 \pm 0.9$ and $ID_{PR} = 3.4 \pm 0.8$ for Go and Anti; $ID_{PR} = 1.9 \pm 0.4$ and $ID_{PR} = 1.9 \pm 0.4$ for Dly-Go and Dly-Anti; $ID_{PR} = 1.13 \pm 0.08$ and $ID_{PR} = 1.1 \pm 0.1$ for RT-Go and RT-Anti). For the RT and Dly tasks, the PR often yields estimates close to 1 ($1 \leq ED_{PR} \leq 2$). This is a direct consequence of the presence of a 'dominant' PC explaining a large fraction of the total variance. While the manifold is certainly stretched across this direction (the cone 'axis' representing time-within-trial), it cannot be really embedded in a one-dimensional space, lest the angular information (which is essential to solve the task) be completely lost. For tasks in the DM family, PR yields $2 \leq ID_{PR} \leq 5$, with variable estimates depending on the training instance. This suggests that, when considering only the strongest PCs, the manifolds are contained within a linear subspace of dimension ≤ 5. For tasks in the MS family, PR yields estimates close to 1, again reflecting the presence of a single dominant PC. Notably, while the range of values is somewhat similar, ID_{PR} and ID_{lFCI} are not correlated (Fig S4E), implying that they capture different geometrical properties: the dimensionality of ID_{PR} captures global variation along different orthogonal axes, the ID_{lFCI} captures local variation.

In Fig S4C we show the results of computing the 'neuronal task complexity' (NTC) metric. Gao et al. [36, 37] proved with a general theoretical argument that NTC is an *upper bound* on the linear dimensionality of neural manifolds given by PR.

In Fig S4D, we show the results of using the multiscale version of the two-nearest-neighbor method. According to [46], multiscale ID estimates can be obtained by progressively 'decimating' the dataset, randomly selecting $100 \cdot 2^K$ points, with $K = 1, \ldots, \log_2(P/100)$ and estimating the ID only on the selected points. For each K, the random choice can be repeated M times, and the average of the M estimates is kept as an estimate of the ID at the scale K. Note that the logic is similar to that of the multiscale FCI method, but the role of K is reversed. Two-NN is intrinsically local, as it considers the distances of the first two neighbors from each point. When using the whole dataset, we are considering the data at the *finest* spatial scale, while small values of K correspond to *coarse* spatial scales - the opposite of what happens for lFCI. As a robust ID estimate, one can consider the coarsest ID obtained with significant decimation of the points ($K = 0$), as estimates obtained for large K are often very sensitive to small-scale noise, which tends to inflate ID estimates [46].
