# Supplementary material for: Exploring neural manifolds across a wide range of intrinsic dimensions
Source: PLoS Comput Biol. 2026 Apr 3;22(4):e1014162. doi: 10.1371/journal.pcbi.1014162 (PMC13068349; doi:10.1371/journal.pcbi.1014162)
Supplement: S4 Text — (PDF) [file pcbi.1014162.s004.pdf]

S4 Intrinsic dimension of high-dimensional visual responses - additional notes

CVPC and noise removal. Consider a neural population of N units responding to a set of S stimuli, presented for two repetitions $r = 1, 2$. Let $x_i^r(s)$ denote the activity (firing rate) of cell i ($i = 1, \ldots, N$) under the r-th presentation ($r = 1, 2$) of stimulus s ($s = 1, \ldots, S$). Activity splits into a stimulus-related response $x_i^*(s)$ and a noise component $\epsilon_i^r(s)$ with zero-mean and independent for different repetitions (such "noise" accounts for inter-trial variability of responses and it includes any activity component that is unrelated to the stimulus):

$$x_i^{(r)}(s) = x_i^*(s) + \epsilon_i^{(r)}(s), \qquad E[e_i^{(r)}(s)] = 0, \quad E[e_i^{(r)}(s), e_i^{(r')}(s)] = 0, \ \forall r' \neq r$$

Responses to all stimuli for repetition r can be collected into the matrix $X^{(r)} \in \mathbb{R}^{S \times N}$ with $X^{(r)} = X^* + E^{(r)}$ (obvious notation).
As highlighted in [74], the data covariance matrix $\frac{1}{S} X^{(r)T} X^{(r)}$ includes noise-related variance, thus differing from $\frac{1}{S} X^{*T} X^*$ which captures stimulus-related variance.
Given a single repetition $X^{(1)}$, a classical approach to find relevant dimensions in the data would be to perform PCA on $X^{(1)}$. The PC loading matrix $U \in \mathbb{R}^{R \times N}$ ($R = \mathrm{rank}(X^{(1)})$) can be obtained from the singular value decomposition $X_{(1)} = UDV^T$, the PC scores matrix being given by $Y^{(1)} = X^{(1)} U^T$. The variance of PCs is given by the eigenvalues $\boldsymbol{\lambda}^{(1)} = (\lambda_1^{(1)}, \ldots, \lambda_R^{(1)}) = \frac{1}{S} \mathrm{diag}(Y^{(1)})^T Y^{(1)}$ and it is affected by the noise component. Ref [66] introduced a method, called cross-validated PCA (cvPCA), leveraging multiple repetitions to obtain reliable estimates of stimulus-related variance. Consider the cross-covariance between the two repetitions, $\frac{1}{S} X^{(2)T} X^{(1)}$: in the space of the PCs of $X^{(1)}$, this becomes $\frac{1}{S} \mathrm{diag}(Y^2)^T Y^1$. In Ref [22] it was shown that $\boldsymbol{\lambda}^{(12)} = \frac{1}{S} \mathrm{diag} Y^{(2)T} Y^{(1)}$ provide a lower bound to λ_i^*, the eigenvalues of the signal-related covariance matrix: in fact, $\forall K \leq C$, $\sum_{k=1}^{K} \lambda_k^{(12)} \leq \sum_{k=1}^{K} \lambda_k^*$. Moreover, the bound is tight under realistic assumptions on the noise structure. Thus, a linear space accounting for a large fraction of the stimulus-related variance can be determined by finding $\min K : \sum_{k=1}^{K} \lambda_k^{(12)} / \mathcal{V} \geq \alpha$, where $\mathcal{V} = \sum_{k=1}^{R} \lambda_k^{(12)} /$ and projecting on the first K rows of U.

ID Results without noise removal. In Fig. S7 A, we show results of a straight application of ID/ED estimation (where we did not preliminarily remove stimulus-unrelated noise). In the two experiments with 2,800 images, the ED (as estimated via PCA with $\alpha = 0.95$) was $\sim 2,200$. Many of the linear dimensions corresponded to low variance, and the effective linear dimension (as estimated by the participation ratio) was nearly one order of magnitude lower, around ~ 200. TWO-NN yielded inconsistent estimates in the two replications (respectively, ~ 40 and ~ 100). Estimates by lFCI yielded a very large range for the two repetitions, $ID_{lFCI} \in [242, 817]$, and $ID_{lFCI} \in [284, 471]$, with best estimates $ID_{lFCI} \sim 557, ID_{lFCI} \sim 447$, of the same order of magnitude than those of the participation ratio.
Discrepancies between lFCI and Two-NN can be explained as in the main text (they are likely caused by Two-NN underestimation bias, as there is no evidence of curvature - δ remained consistently below 1. The larger discrepancy between the participation ratio and lFCI (as compared to what seen in the main text) is probably due to the fact that stimulus-unrelated noise adds many dimensions with low variance.
Surprisingly, when considering the experiment with 32 natural images, both the ED and the ID remained close to values observed for 2,800 stimuli. PCA yielded an ED $\sim 2,000$, again a reflection of many directions with small variance - and the participation ratio yielded ~ 100. Two-NN estimates were $ID_{TWO-NN} \sim 50$, while lFCI provided

a wide range, $ID_{lFCI} \in [80, 731]$ with best estimate given by $ID_{lFCI} = 399$, much larger than those of TWO-NN. In the experiment with gratings, all dimension estimates dropped, but the decrease was drastic only for the effective linear dimension estimated by the participation ratio, which is ~ 30. The linear dimension estimated with PCA was still large, $\sim 1,050$. Two-NN yielded $ID_{TWO-NN} \sim 75$, $ID_{lFCI} \in [86, 233]$ with best estimate $ID_{lFCI} \sim 133$. The lack of striking differences in the ID of visual responses between experiments with very different stimulus sets suggests that stimulus-unrelated variance contributes dominantly to the manifold's ID in the cases with 32 images.
