# Supplementary figures and images for: Exploring neural manifolds across a wide range of intrinsic dimensions
Source: PLoS Comput Biol. 2026 Apr 3;22(4):e1014162. doi: 10.1371/journal.pcbi.1014162 (PMC13068349; doi:10.1371/journal.pcbi.1014162)

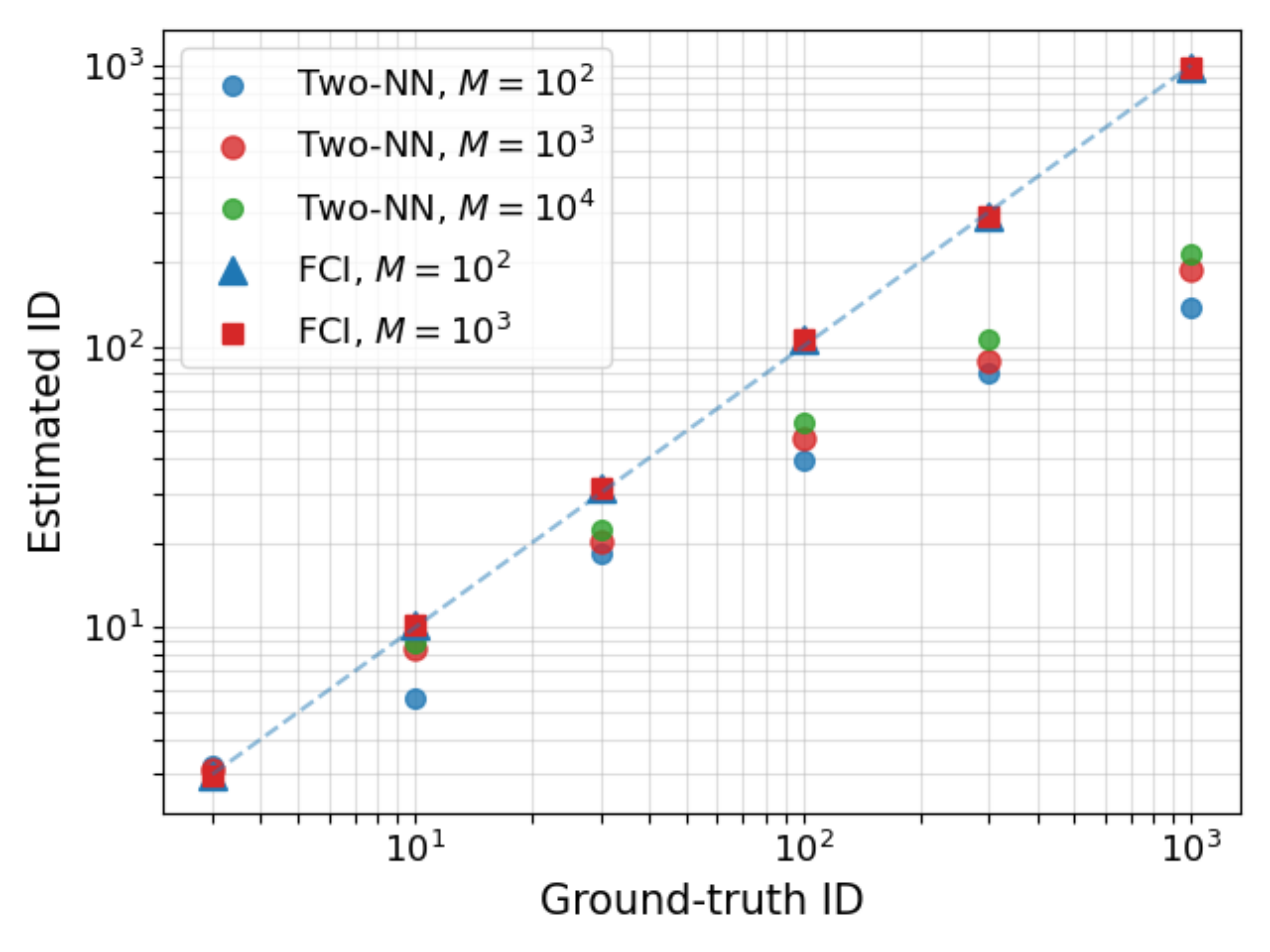

Supplement: S1 Fig — We show results of FCI on M uniformly sampled data (M=102,103,104) on hypercubes of known dimension varying from D = 10 to D = 1000. Results are compared with those of Two-NN, a common ID estimator. (TIF) [file pcbi.1014162.s005.tif]

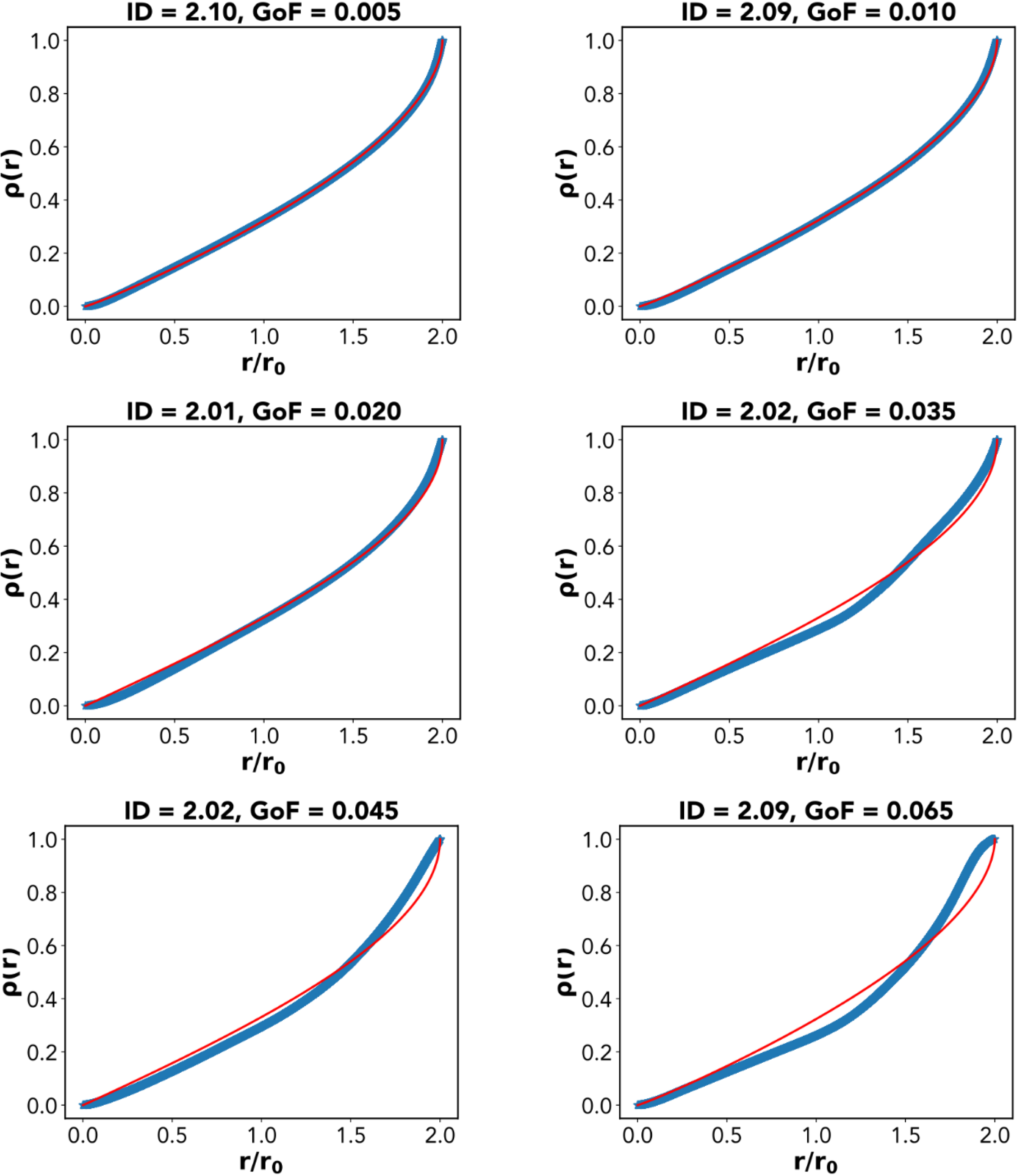

Supplement: S2 Fig — (TIF) [file pcbi.1014162.s006.tif]

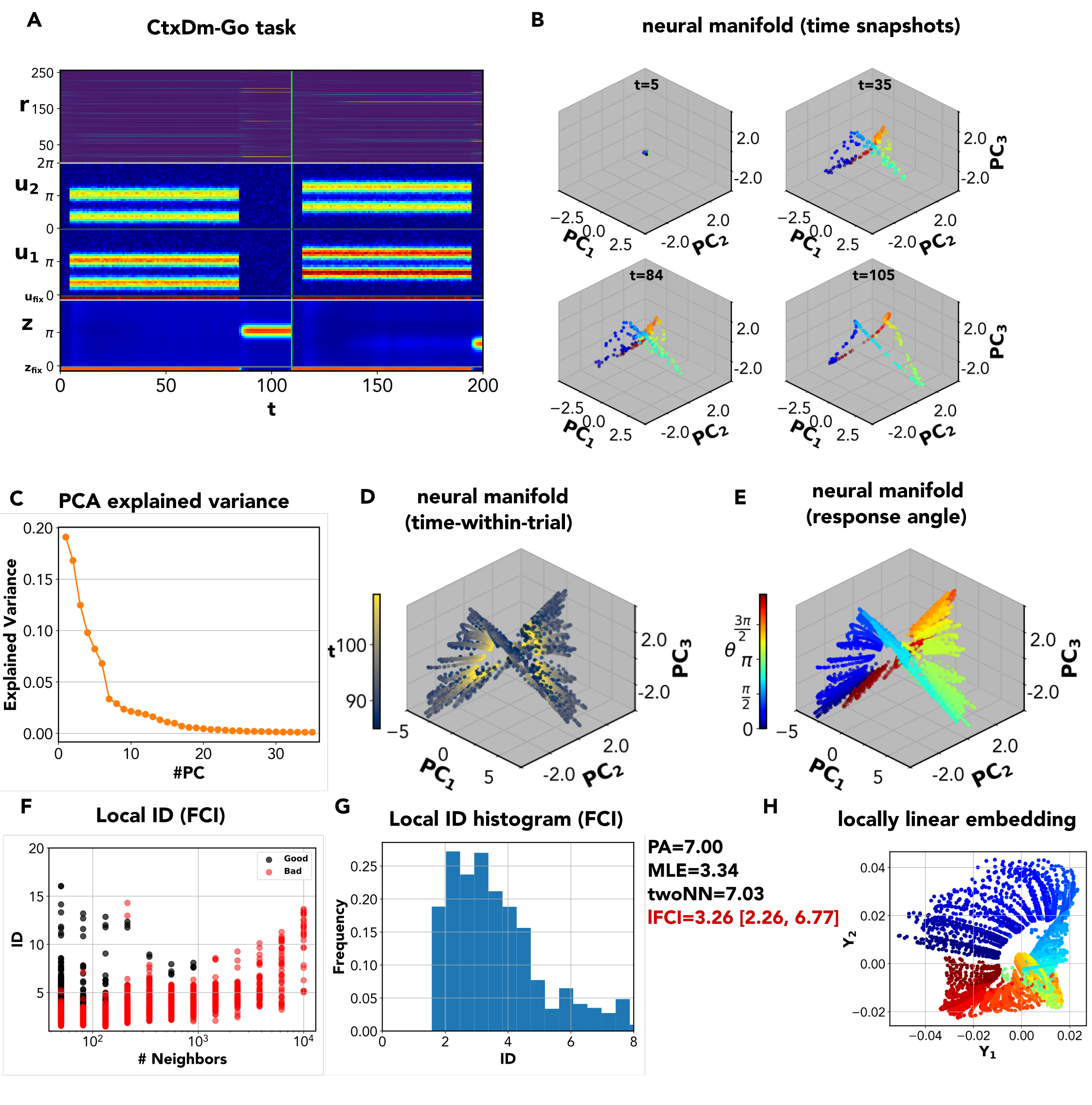

Supplement: S3 Fig — A Input, output and network activity for the Ctx-DM1 task; B Input, output and network activity for the Ctx-DM1 task; C Explained variance of the different PCs of the network activity. Looking at the variance, PCA predicts a dimensionality of 1 if we consider the highest drop in variance or a dimension of 7 if we consider the second elbow in variance. D Projection of the network activity on the first three PCs. The color code stands for time-within-trial (time from trial onset). PC1 is nearly aligned with time-within-trial. E Projection of the network activity on the first three PCs. The color code stands for response angle. F Multiscale ID plot for the RT-Go task. At low K, estimates are close to 3. G Local ID histogram, showing a peak at D = 3.26. H Locally linear embedding can flatten the manifold for the CTx-DM1 task onto a 2-D surface but only with some approximation. (TIF) [file pcbi.1014162.s007.tif]

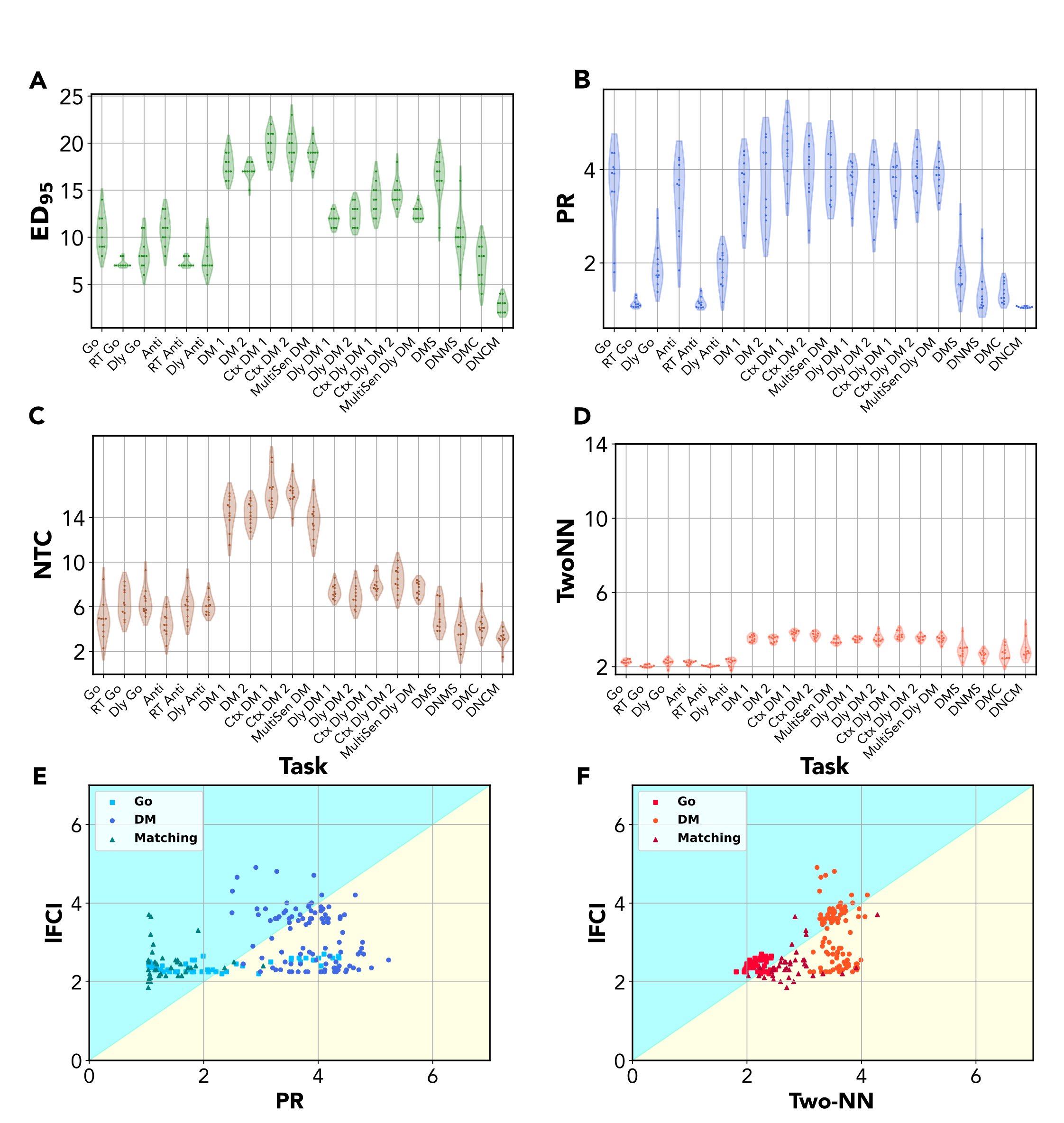

Supplement: S4 Fig — For each task in the Cog-Tasks battery, we trained 10 independent RNNs to solve the task and computed the ID of the neural activity manifolds for each case. (A) ID as the number of PCs identified with the principal component analysis (PCA) keeping the first principal components explaining 95% of the variance (B) ID estimated by the participation ratio (PR) (C) upper bound on the ID given by neuronal task complexity (NTC) (D) ID computed through the multiscale (with decimation) Two-NN method. (E) NTC can under- and overestimate the ID (F) The ID identified by the multiscale Two-NN method and the local FCI are in good agreement. (TIF) [file pcbi.1014162.s008.tif]

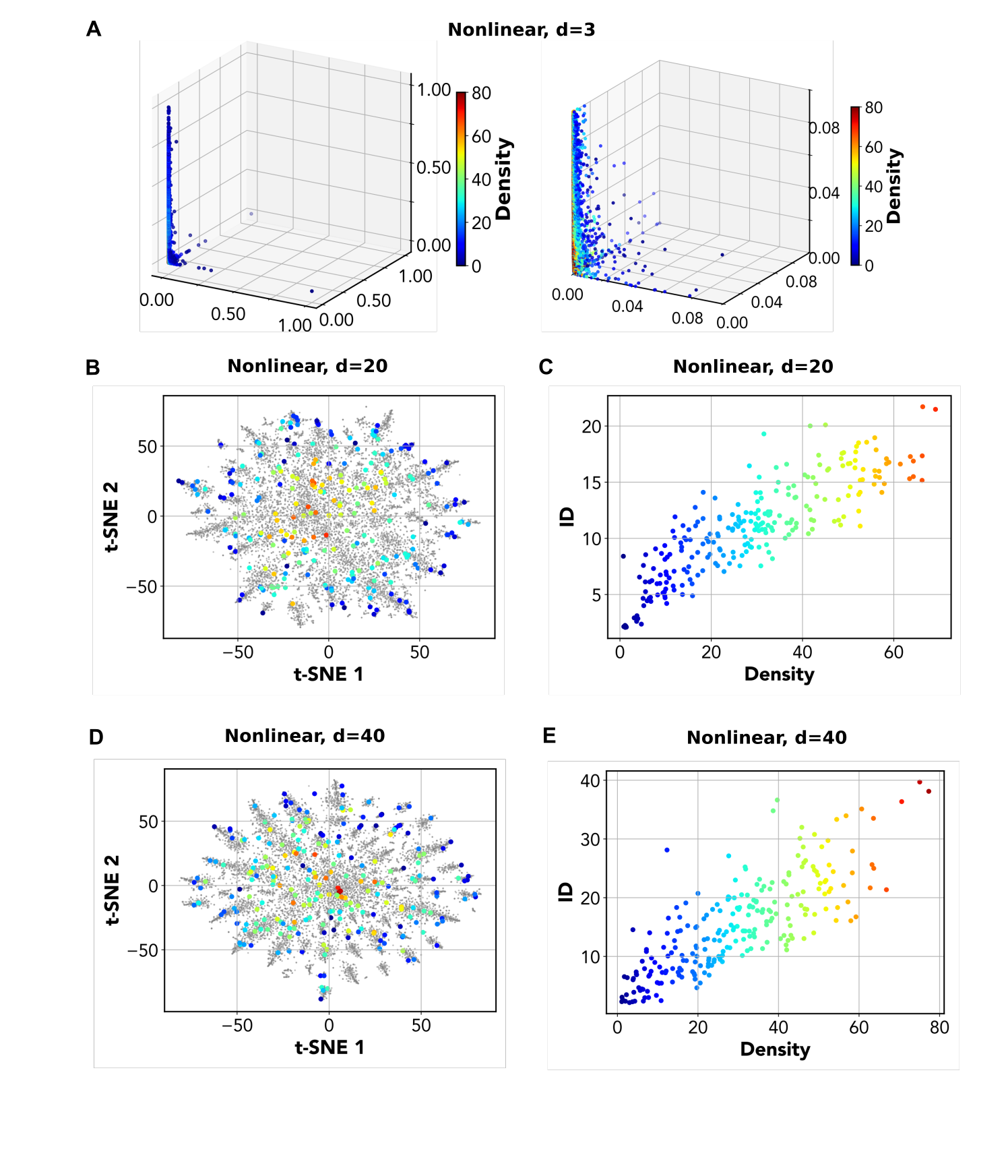

Supplement: S5 Fig — (A) TSNE embedding of the data with d = 20. Low local IDs are found in the ‘periphery’, while high local IDs are found in the ‘core’ (B) local IDs estimates strongly correlate with the local density of points for d = 20. (C) same as (A) for d = 40. (D) local IDs estimates strongly correlate with the local density of points for d = 20. (TIF) [file pcbi.1014162.s009.tif]

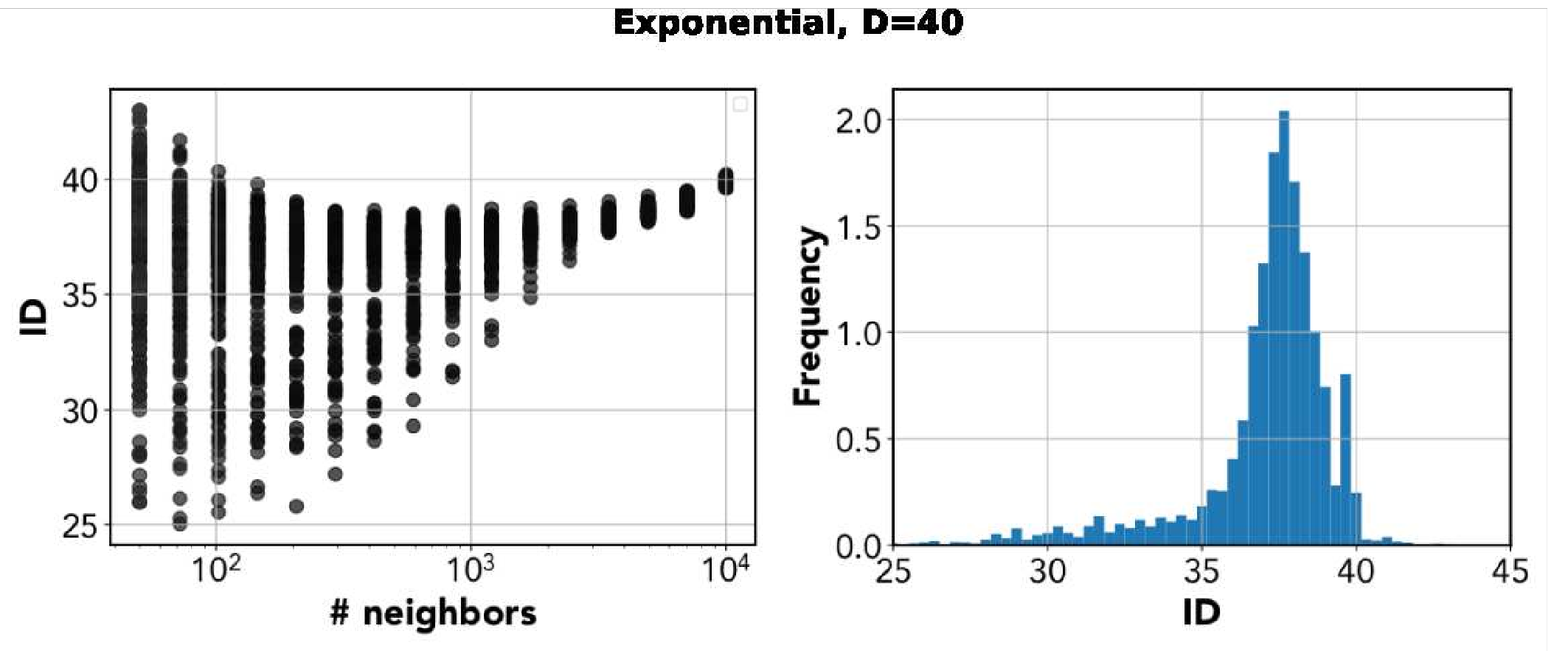

Supplement: S6 Fig — (TIFF) [file pcbi.1014162.s010.tiff]

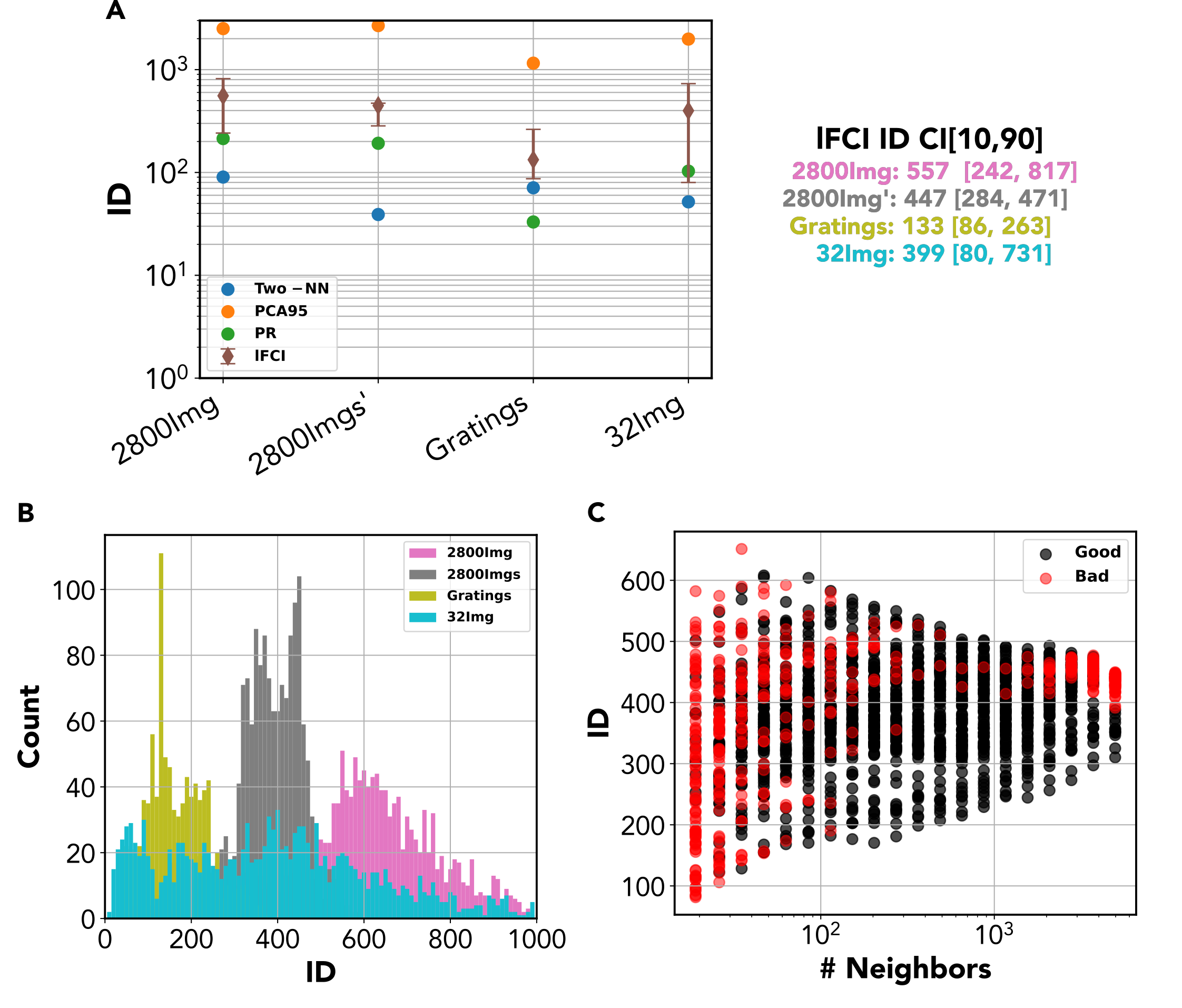

Supplement: S7 Fig — Results of the raw high dimensional visual responses recorded by Stringer [67]. A ID and ED values for the different experiments; B lFCI ID distribution for the different experiment. C ID as a function of the number of neighbors for the experiment with 2,800 images. (TIF) [file pcbi.1014162.s011.tif]

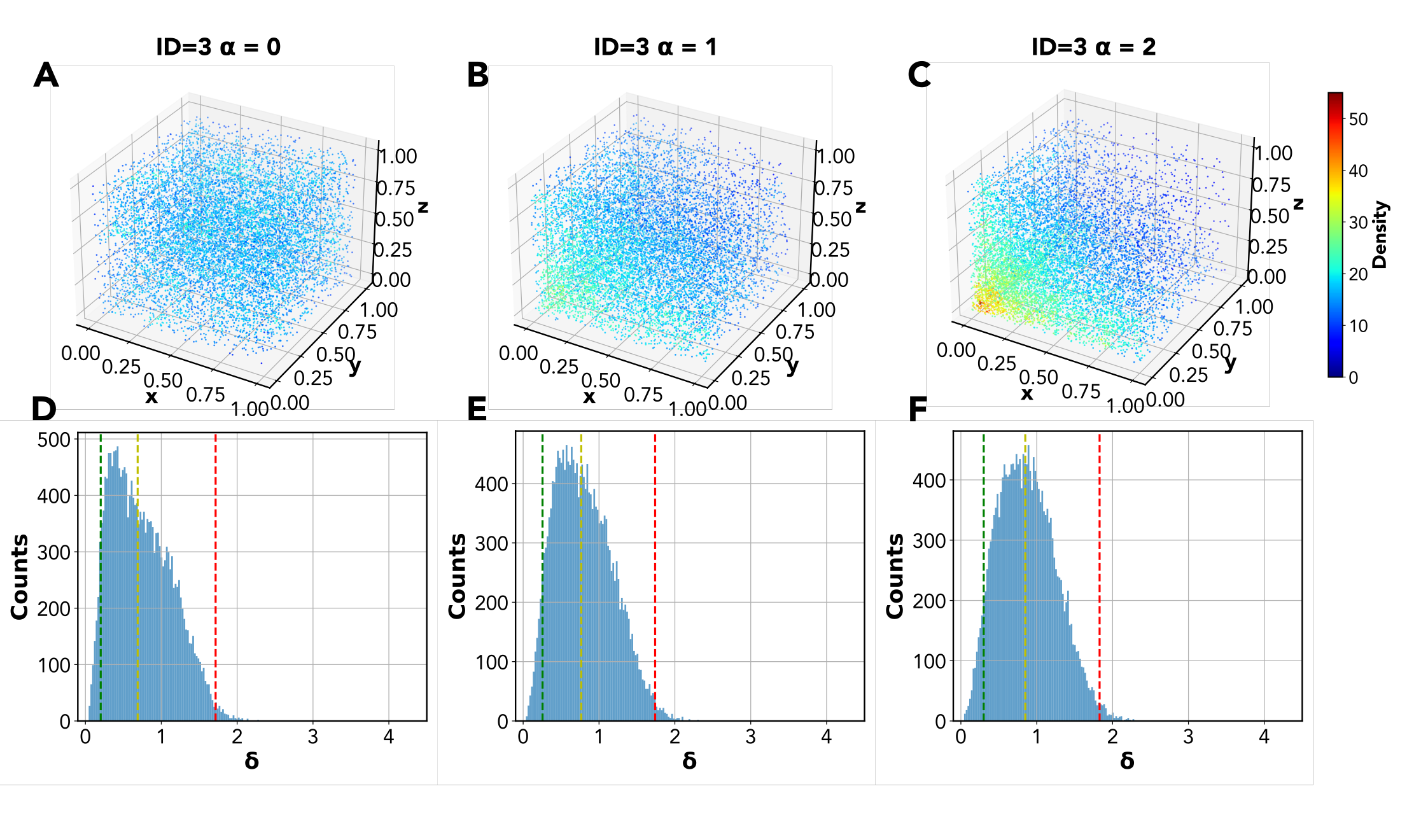

Supplement: S8 Fig — Distribution of the values of for three different dataset with increasing anisotropy and fixed ID = 3. (A), α=0 corresponds to an isotropic distribution. (B, C), α=1 and α=2 correspond to non isotropic distributions as points tend to accumulate around the origin and the axes. The data for α=2 are already strongly non-isotropic as they display a strong density gradient. For each data set, we considered local neighborhoods of varying size K and computed δ and GoF. In panels D-F, we show the resulting distributions of δ for ID = 3. The distributions are only mildly affected by α and the values remain strictly bounded. In particular, the 99-th percentile of the δ distribution is always lower than δ=2 (red dashed line in the plots). (TIFF) [file pcbi.1014162.s012.tiff]

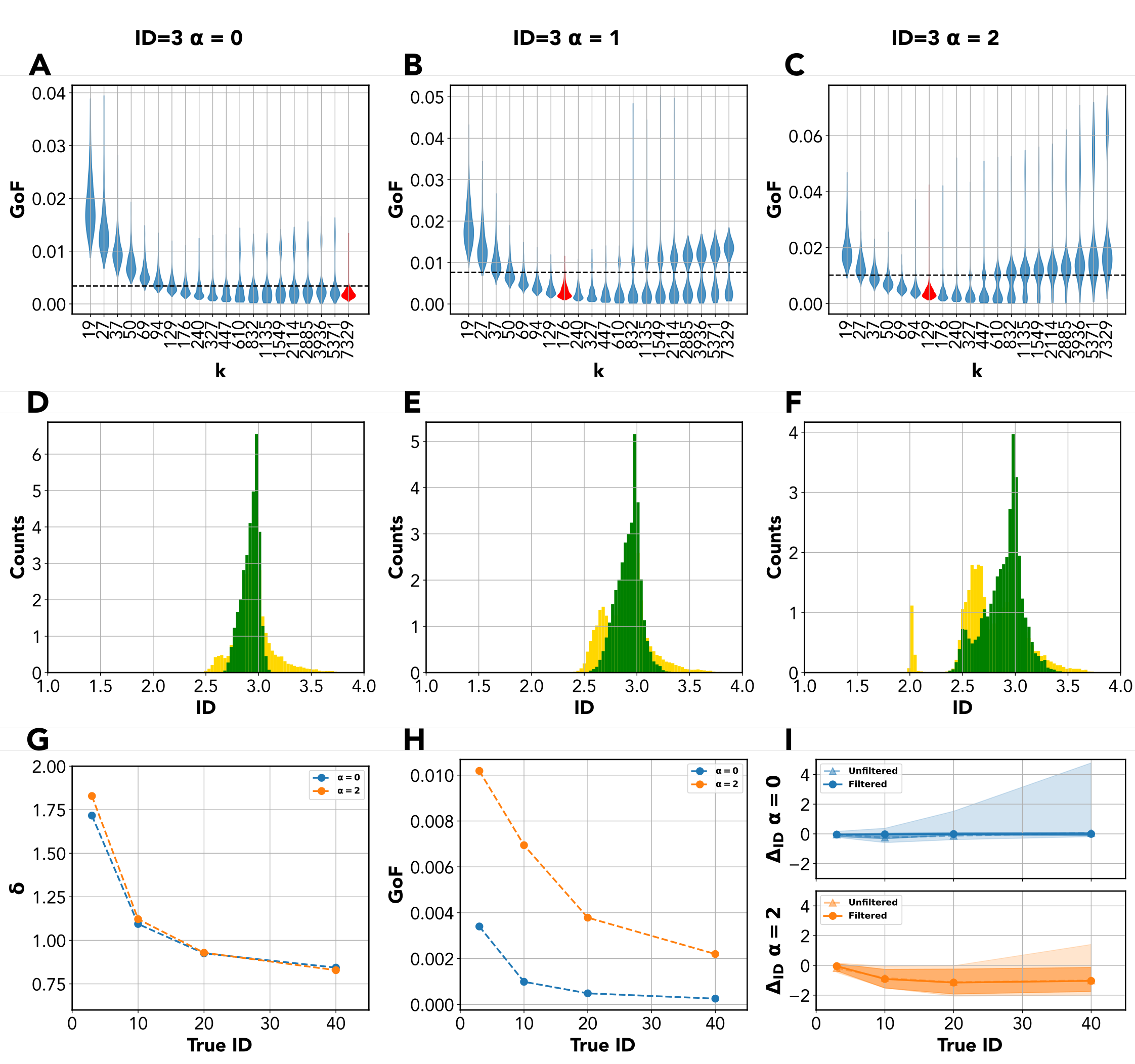

Supplement: S9 Fig — A-C show the distributions of GoF, grouped according to neighborhood size K. In this case, the distributions are strongly affected by α; in red the distribution with the lowest median. For α = 0 the largest neighborhoods yield the most consistent GoF distribution. For α = 1, the distribution becomes clearly bimodal for all K ≥ 1000; For α = 2, the effects of anisotropy are so strong that most large neighborhoods (K ≥ 1000) yield GoF > 0.01. In this case, the neighborhoods yielding the most consistent GoF distribution correspond to K ≈ 150. (D-F) ID distributions for the different, in yellow the distribution for all the estimates, in green the distribution of ID after the filtering process. To ensure that analogous results could be obtained in a wide range of dimensions we repeated the same analysis for D = 3, D = 10, D = 20, D = 40. (G) the 99-th percentile of the distribution, that always remains below δ = 2. (H) the 99-th percentile of the GoF distribution corresponding to the K yielding the lowest median GoF. We see that the GoF threshold is highly variable from dataset to dataset. In all cases, removing ‘bad’ estimates improved the ID estimate (I). (TIF) [file pcbi.1014162.s013.tif]

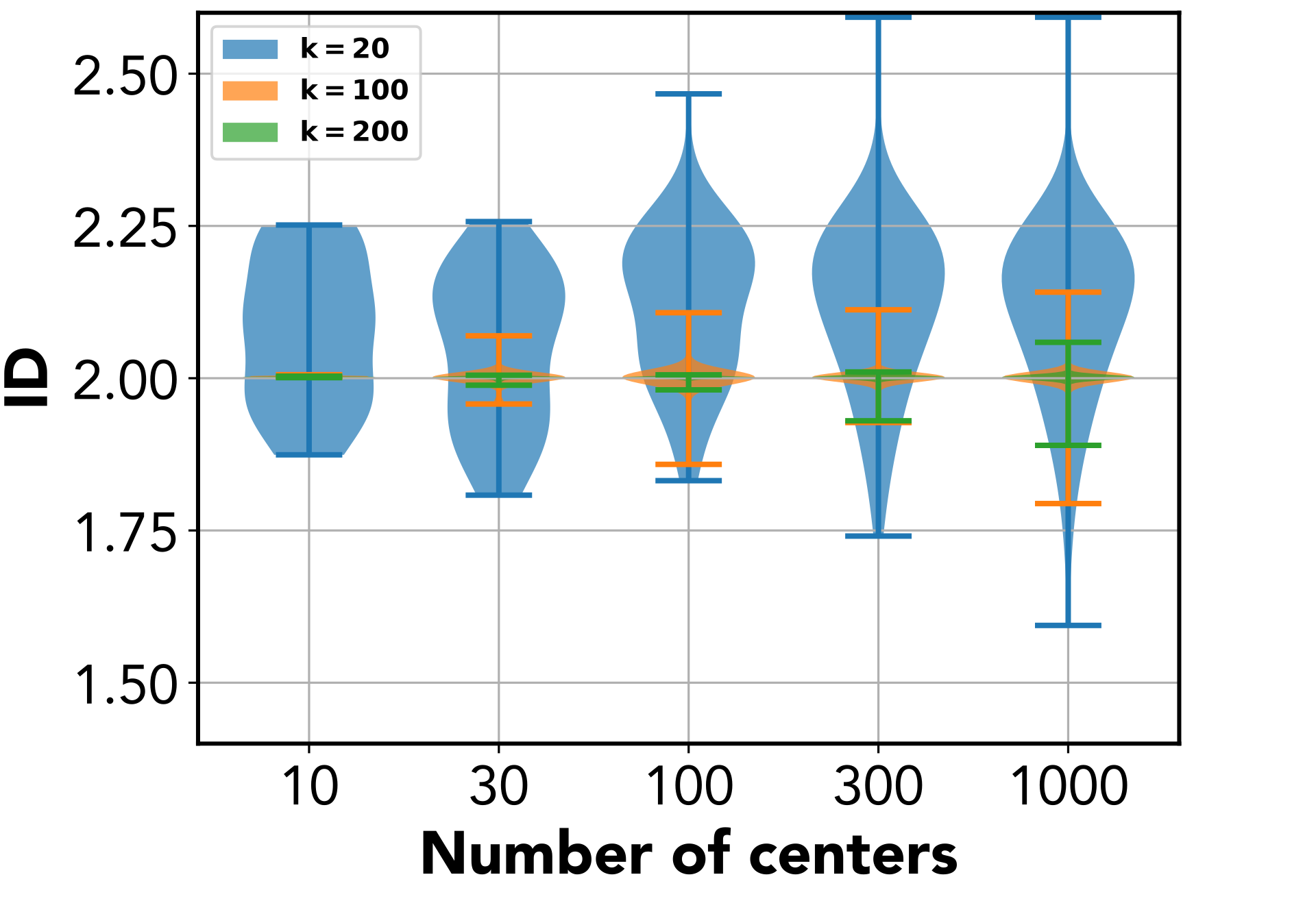

Supplement: S10 Fig — For the Swiss Roll, we computed the local ID histogram varying the number of centers M = 10,30,100,300,1000. The number of centers affects only marginally the distribution of the ID estimates. The size of the neighborhoods is the parameter that most affects the estimation. (TIF) [file pcbi.1014162.s014.tif]
